# Comparative Analysis of Breastfeeding and Infant Formulas: Short‐ and Long‐Term Impacts on Infant Nutrition and Health

**DOI:** 10.1002/fsn3.70788

**Published:** 2025-08-27

**Authors:** Kalmee Pramoda Kariyawasam, Geeshani Somaratne, Sumali Dilrukshi Dillimuni, Umani Walallawita

**Affiliations:** ^1^ Riddet Institute Massey University Palmerston North New Zealand; ^2^ Postgraduate Institute of Agriculture University of Peradeniya Peradeniya Sri Lanka; ^3^ Department of Food Science and Technology, Faculty of Agriculture University of Peradeniya Peradeniya Sri Lanka; ^4^ Research Operations Massey University Palmerston North New Zealand

**Keywords:** alternative, dynamic composition, early childhood, feeding recommendations, growth and development

## Abstract

Infant nutrition plays a pivotal role in shaping short‐ and long‐term health outcomes. This review critically examines the comparative effects of breastfeeding and infant formulas on infant growth, cognitive development, and overall health. Breastfeeding, often considered the gold standard, provides a dynamic composition of nutrients, antibodies, and bioactive factors that support immune function, neurodevelopment, and gut microbiota maturation, contributing to long‐term protection against chronic diseases, such as obesity, type 2 diabetes, and cardiovascular conditions. In contrast, although modern infant formulas are designed to mimic the nutritional composition of breast milk and support infant growth, they may lack certain bioactive components, potentially leading to differences in gut microbiota composition and increased susceptibility to infections and allergies. However, various medical, societal, and personal factors may necessitate formula feeding, which has been optimized to approximate the nutritional profile of breast milk. In conclusion, both breastfeeding and infant formulas play significant roles in infant nutrition, with breastfeeding providing unparalleled health benefits. However, infant formulas remain a viable alternative when breastfeeding is not possible. This review highlights the need for individualized feeding recommendations that consider maternal health, socioeconomic factors, and infant needs to optimize growth and health outcomes.

## Graphical abstract

Breastfeeding provides various benefits, such as supporting the immune system and promoting brain and physical development. Infant formulas are a viable alternative to support growth when breastfeeding is not feasible. The review emphasizes that both methods are important and calls for personalized feeding guidance based on the mother's health, living conditions, and available resources. ADHD, attention deficit hyperactivity disorder; ARA, arachidonic acid; ASD, autism spectrum disorder; DHA, docosahexaenoic acid; HMO, human milk oligosaccharides; IQ, intelligence quotient.

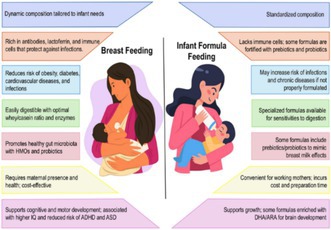

## 1 Introduction

Infancy represents a pivotal stage in human development, setting the stage for lifelong health and well‐being. It is typically defined as the period from birth to 12 months of age, and it is a time of unparalleled growth and development (Victora et al. 2016; Deoni 2018). During this critical period, infants undergo rapid physical, cognitive, and emotional transformations (Berger et al. 2020). The brain, for instance, experiences significant growth, with synaptic connections forming at an astonishing rate, laying the foundation for cognitive and motor skills development (Deoni 2018). Furthermore, organ systems mature and functionalize, preparing the infant for the diverse challenges of the external environment (Victora et al. 2016). The nutritional decisions made during this critical period have significant implications for immediate health outcomes and long‐term growth and development (Martín‐Rodríguez et al. 2022). Among the various feeding options available, breastfeeding and infant formulas emerge as the primary methods of infant nourishment (Martin et al. 2016). The importance of optimal nutrition during infancy cannot be overstated, given its profound impact on a range of health outcomes throughout an individual's life.

Global data from 2020 present alarming figures: 149 million children under the age of five suffered from stunted growth, 45 million experienced wasting, and 38.9 million were affected by overweight or obesity (World Health Organization 2021). Undernutrition was directly linked to 45% of child fatalities, highlighting the urgent need for effective and sustainable infant‐feeding strategies to improve global health outcomes. Breast milk, a complex and dynamic biological fluid, is uniquely and readily available to meet the nutritional needs of infants (Victora et al. 2016; Martín‐Rodríguez et al. 2022). Its composition is designed to evolve in alignment with the changing requirements of the growing baby and is enriched with bioactive components that strengthen immune defenses and overall health (Victora et al. 2016; Hanson and Winberg 2016). Moreover, breastfeeding facilitates the establishment of a diverse gut microbiome, which is vital for immune system maturation and metabolic balance (Backhed et al. 2015).

A plethora of research underscores the manifold benefits of breastfeeding, encompassing both immediate and long‐term advantages. In addition to supplying vital nutrients, breast milk offers protection against infections, reduces the risk of sudden infant death syndrome, and promotes mother–infant (Victora et al. 2016; Hanson and Winberg 2016). Longitudinal studies have also associated breastfeeding with enhanced cognitive outcomes, reduced risks of attention deficit/hyperactivity disorder, and lower incidences of autism spectrum disorder (Anderson, Vaillancourt, et al. 2016; Victora et al. 2015). Despite the undeniable benefits of breastfeeding, it may not always be a feasible or preferred option for every family. In such instances, infant formulas, meticulously designed substitutes, aim to deliver essential nutrients to infants. These formulas, primarily derived from cow's milk or soymilk, strive to emulate breast milk's nutritional profile (Koletzko et al. 2019). Although significant advancements have been made in formula composition and production techniques, questions persist regarding their ability to fully replicate the unique attributes of breast milk, such as bioavailability and immunological factors (Hanson and Winberg 2016).

This review seeks to critically evaluate existing research and synthesize findings to provide a comprehensive understanding of the comparative effects of breastfeeding and infant formulas. The study systematically synthesized recent peer‐reviewed studies (2015–2025) on the nutritional, developmental, and health impacts of breastfeeding and infant formula. Both observational and interventional research, including randomized trials and meta‐analyses, were thematically analyzed to compare short‐ and long‐term outcomes. The review's primary objectives include examining short‐term nutritional and health outcomes, assessing long‐term cognitive and neurodevelopmental impacts, evaluating various health outcomes, and exploring socioeconomic and cultural factors influencing infant‐feeding choices. By addressing these objectives, the review aimed to offer an in‐depth assessment of the influences of breastfeeding and infant formulas on infant growth, development, and overall health.

## 2 Understanding the Complexities of Breast Milk Production Mechanisms and Influencing Factors

### 2.1 Preparing Breasts for Lactation

**FIGURE 1:**
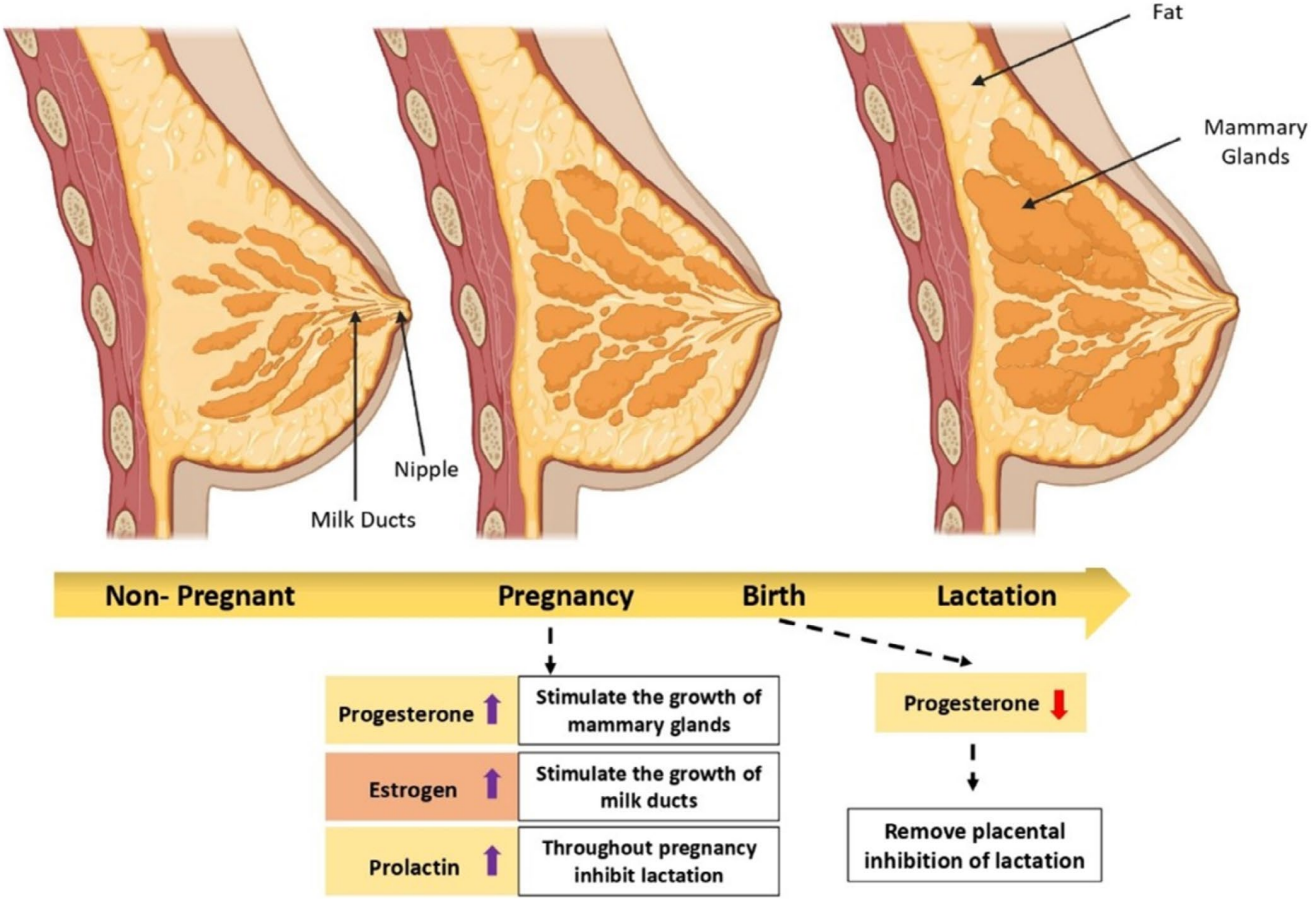
Preparing the breast for breast milk production and breastfeeding.

Preparing the breast for breastfeeding is essential to ensure a smooth and comfortable nursing experience for both mother and baby. Proper preparation helps in establishing a good latch, ensuring adequate milk supply, and preventing complications, such as sore nipples or mastitis (Bekuma and Galmessa 2018). One of the first steps in preparing the breast is understanding the anatomy and physiology of lactation. The breast is composed of glandular tissue, which produces milk, and ducts that transport the milk to the nipple (Haider 2023). As seen in Figure 1, during pregnancy, hormonal changes prepare the breast for lactation by increasing the number and size of milk‐producing cells, known as alveoli, and expanding the milk ducts (Chowdhury et al. 2015; Dai et al. 2022). To prepare the breast for breastfeeding, it is important to maintain good hygiene (Bekuma and Galmessa 2018). Washing the nipples with water only and avoiding harsh soaps or cleansers can help prevent dryness and irritation. Additionally, keeping the nipples dry and allowing them to air dry after feeding or expressing milk can help prevent cracking and soreness (Bekuma and Galmessa 2018; Pommeret‐de et al. 2022).

Another crucial aspect of preparing the breast for breastfeeding is practicing proper latch and positioning techniques. A good latch is essential for effective milk transfer and to prevent nipple pain and damage. Mothers should ensure that the baby's mouth covers both the nipple and a significant portion of the areola to facilitate proper suction and milk flow (Amir et al. 2021; Pehlivan and Bozkurt 2021). Various breastfeeding positions, such as the cradle hold, football hold, or side‐lying position, can be explored to find the most comfortable and effective position for both mother and baby (Pehlivan and Bozkurt 2021). In addition to preparing the breast physically, it is important to prepare for breastfeeding mentally and emotionally. Education and support from healthcare professionals, lactation consultants, and support groups can be invaluable in building confidence and addressing concerns or challenges that may arise (Rempel et al. 2017). Developing a support network of family and friends who can offer encouragement and assistance can also be beneficial in navigating the breastfeeding journey. Lastly, maintaining a healthy lifestyle can contribute to optimal breastfeeding outcomes. Eating a balanced diet rich in nutrients, staying hydrated, and getting adequate rest can support milk production and overall well‐being (Ford et al. 2020). Avoiding smoking, alcohol, and certain medications that can interfere with milk production or harm the baby is also important (Ford et al. 2020; Keikha et al. 2017).

**FIGURE 2:**
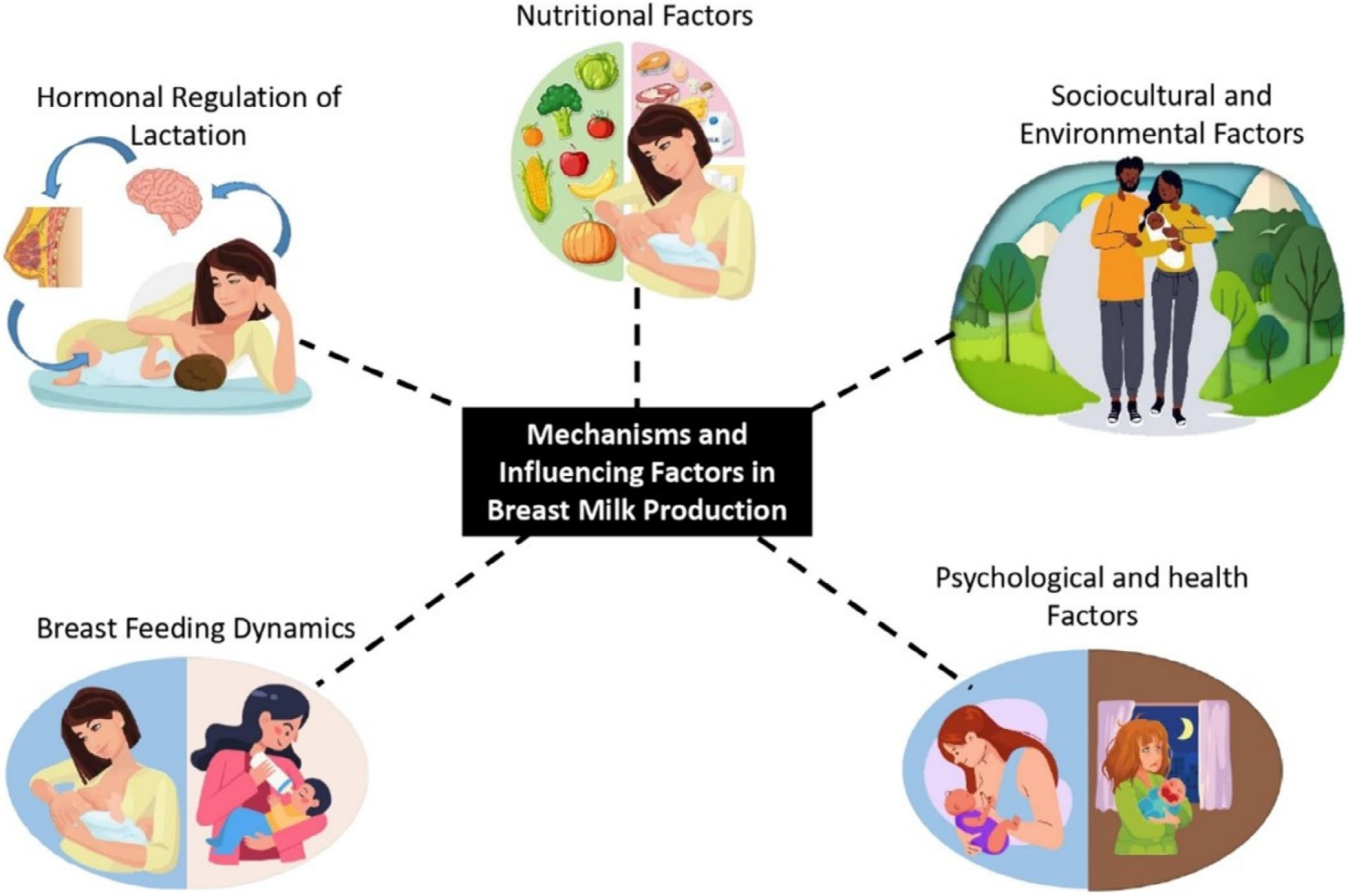
Mechanisms and influencing factors in breast milk production.

Breast milk production, known as lactation, is a multifaceted and well‐coordinated physiological process governed by a myriad of mechanisms and influenced by a range of factors (Figure 2).

### 2.2 Hormonal Regulation of Lactation and Breastfeeding Dynamics

**FIGURE 3:**
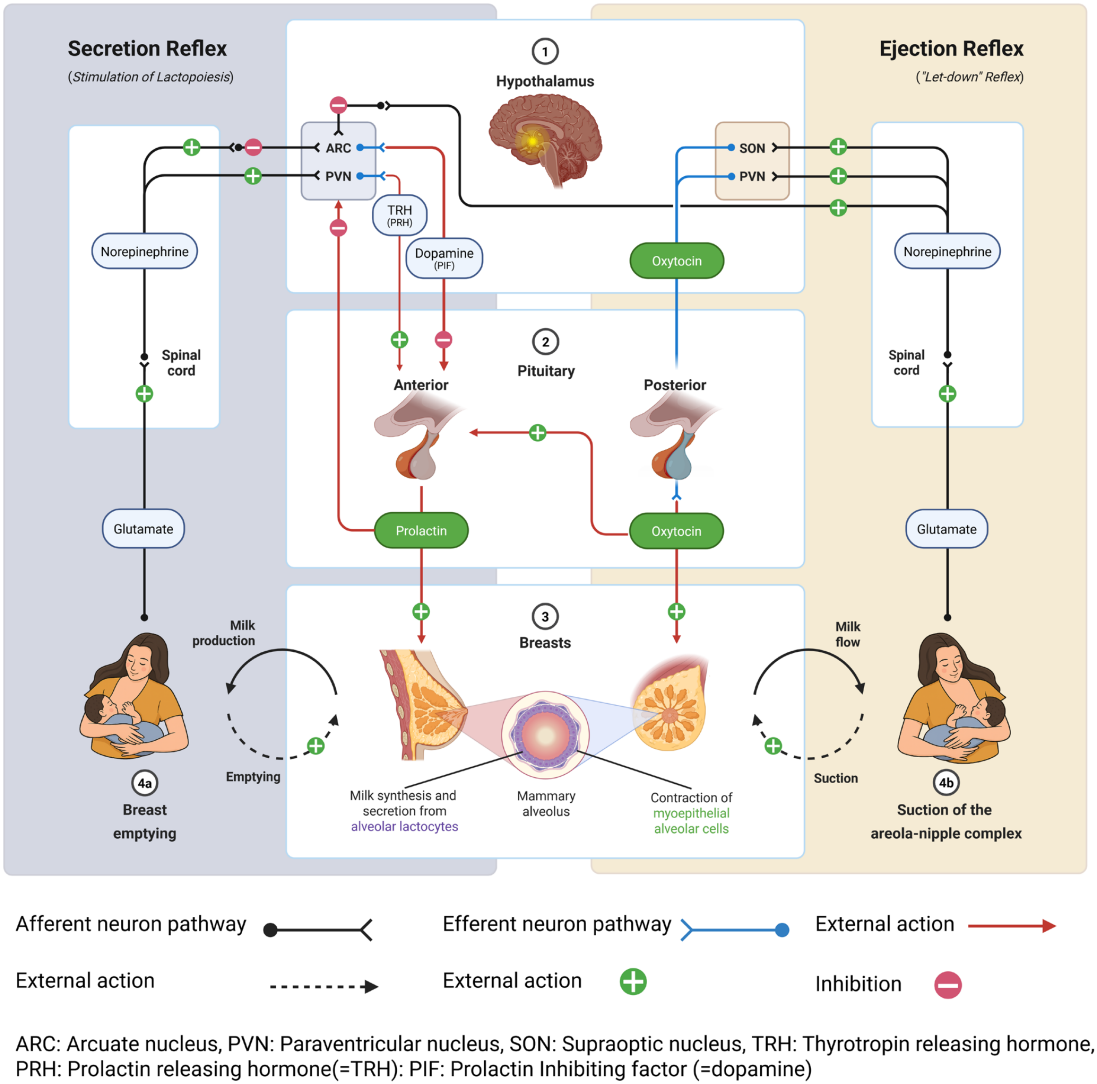
Hormonal regulation of lactation: Roles of prolactin and oxytocin.

The efficiency of milk removal from the breast is intrinsically linked to milk yield. Regular breastfeeding or breast pumping is essential in maintaining lactation feedback loops and stimulating ongoing milk production (Innis 2014). The frequency, duration, and effectiveness of breastfeeding sessions significantly influence milk production and supply (Chowdhury et al. 2015; Phillipps et al. 2020). Figure 3 visually represents the complex interplay between prolactin and oxytocin in the hormonal regulation of lactation. This complex hormonal regulation is essential for the nourishment and growth of newborns and plays a pivotal role in the mother–infant bonding process (Innis 2014; Ivanova et al. 2021). Central to lactation are hormonal regulators, predominantly prolactin and oxytocin, coordinating the synthesis and ejection of milk, respectively (Innis 2014). Prolactin, primarily secreted by the anterior pituitary gland, functions in stimulating the development and proliferation of mammary glandular tissue. It plays a fundamental role in initiating and maintaining milk synthesis, ensuring that the mammary glands are adequately prepared to produce milk postpartum (Chowdhury et al. 2015). The release of prolactin is not constant but rather pulsatile, and its secretion is primarily controlled by the hypothalamic hormone, dopamine (Phillipps et al. 2020). Breast stimulation or suckling triggers the release of prolactin, setting in motion a series of physiological events leading to milk production (Ivanova et al. 2021). The role of prolactin extends beyond just milk production; it also has various physiological functions that support lactation. Prolactin promotes the differentiation of mammary epithelial cells, ensuring optimal milk composition and quality (Chowdhury et al. 2015; Phillipps et al. 2020). Moreover, prolactin acts synergistically with other hormones, such as insulin and cortisol, to regulate metabolic processes during lactation, ensuring that the mother has adequate energy reserves to sustain milk production (Paragliola et al. 2022).

Conversely, oxytocin, released from the posterior pituitary gland, plays a pivotal role in the milk ejection reflex, commonly known as the let‐down reflex (Ivanova et al. 2021). Oxytocin targets the myoepithelial cells enveloping the alveoli, inducing their contraction and facilitating milk ejection into the ducts (Dai et al. 2022). The release of oxytocin is intricately linked to the emotional and psychological state of the mother. Positive interactions with the infant, such as breastfeeding and cuddling, stimulate the release of oxytocin, promoting feelings of relaxation, bonding, and maternal attachment (Moberg et al. 2020). Oxytocin is often referred to as the “love hormone” or “bonding hormone” due to its role in facilitating social bonding and emotional attachment between the mother and infant (Dai et al. 2022; Moberg et al. 2020). The interplay between prolactin and oxytocin is crucial for the successful initiation, maintenance, and cessation of lactation. While prolactin stimulates milk production, oxytocin ensures effective milk ejection, ensuring that the infant receives adequate nutrition for growth and development. This hormonal regulation is dynamic, adapting to the changing needs of the mother and infant throughout the lactation period (Ivanova et al. 2021).

### 2.3 Nutritional Factors

Maternal nutrition plays an indispensable role in supporting lactation, acting synergistically with hormonal cues to optimize milk synthesis and volume. A balanced diet that includes adequate calorie intake, hydration, and a balanced nutrient profile is vital for sustaining optimal milk production (Ford et al. 2020; Wambach and Riordan 2015). Protein stands out as a crucial nutrient for lactating mothers, supporting tissue repair and muscle growth. Studies indicate that incorporating protein‐rich foods like lean meats, poultry, fish, eggs, dairy products, legumes, and nuts can significantly boost breast milk production (Bravi et al. 2016). These food sources are replete with essential amino acids, fulfilling the heightened protein demands of lactating mothers and ensuring adequate milk synthesis. Diversifying protein sources in the maternal diet not only supports optimal milk production but also contributes to infant growth and development (Ford et al. 2020). Omega‐3 fatty acids, particularly docosahexaenoic acid and eicosapentaenoic acid, are fundamental for infant brain and eye development (Keikha et al. 2021). Research underscores the importance of maternal consumption of omega‐3‐rich foods, such as fatty fish (salmon, mackerel, and sardines), flaxseeds, chia seeds, and walnuts in augmenting the levels of these critical fatty acids in breast milk (Verduci, Giannì, and Benedetto 2020). A diverse intake of fruits and vegetables amplifies the nutrient content of breast milk, delivering essential vitamins like vitamin C, vitamin A, folate, and potassium (Boran and Aktac 2018). Calcium is indispensable for bone health and muscle function, making it a key nutrient during lactation. Research underscores the significance of consuming calcium‐rich foods, such as dairy products (milk, yogurt, and cheese), leafy greens (kale and collard greens), tofu, and fortified foods to ensure adequate calcium intake during lactation (Jung et al. 2016). Maternal hydration status plays a critical role in determining milk volume, with inadequate hydration potentially leading to decreased milk supply (Bzikowska‐Jura et al. 2018). Although specific fluid intake recommendations for lactating mothers remain inconclusive, research advocates for drinking to thirst and ensuring a consistent intake of fluids throughout the day, encompassing water, herbal teas, and milk (Ryan et al. 2022). Monitoring hydration cues and responding appropriately can safeguard optimal breast milk production and overall maternal well‐being.

### 2.4 Psychological and Health Factors

Psychological well‐being, encompassing maternal stress levels and emotional states, plays a key role in lactation. Elevated stress or anxiety levels can impede milk let‐down and hinder optimal milk production, underscoring the intertwining of physiological and psychological aspects in lactation (Ford et al. 2020; Wambach and Riordan 2015; Dewi 2023). Concurrently, underlying health conditions like hormonal imbalances, thyroid disorders, and specific medications can detrimentally affect both milk production and composition (Dewi 2023; Septianingrum et al. 2020). Addressing these health concerns is paramount to ensuring optimal lactation and adequate milk supply.

### 2.5 Environmental and Sociocultural Influences

Environmental and sociocultural factors also wield significant influence over breast milk production. Access to adequate breastfeeding support and lactation resources is pivotal, with informed and supported mothers more likely to establish and sustain successful breastfeeding relationships (Chowdhury et al. 2015). Moreover, cultural beliefs and societal attitudes toward breastfeeding can shape maternal breastfeeding practices, subsequently impacting milk production and infant‐feeding choices (Rempel et al. 2017; Ivanova et al. 2021). Cultivating supportive environments that promote breastfeeding and offer robust lactation support can be helpful in strengthening breastfeeding outcomes and fostering improved maternal–infant health (Modak et al. 2023).

## 3 Breast Milk Composition and Its Unique Functional Properties for Infant Growth and Development

Colostrum is the first milk produced by the mammary glands during the initial days after childbirth (Pandey et al. 2021). This unique fluid plays a vital role in providing newborns with essential nutrients, antibodies, and bioactive compounds necessary for early development and immune system priming (Godhia and Patel 2013). Table 1 presents a comparative analysis of the composition of human milk colostrum, characterized by its concentrated levels of proteins, vitamins, and minerals, making it a nutritional superfood for newborns. Although it may appear thicker and yellower than mature breast milk, colostrum is low in fat and carbohydrates but rich in proteins, including immunoglobulins, lactoferrin, and growth factors (Pandey et al. 2021). One of the most significant attributes of colostrum is its high concentration of antibodies, particularly immunoglobulin A (IgA) (Verduci, Giannì, and Benedetto 2020; Bzikowska‐Jura et al. 2018; Godhia and Patel 2013). These antibodies are vital for providing passive immunity to the newborn, offering protection against a wide range of pathogens during the initial days of life when the infant's immune system is still developing (Keikha et al. 2021; Hanson and Winberg 2016). In addition to antibodies, colostrum contains a myriad of bioactive compounds and growth factors that play crucial roles in promoting gut maturation, enhancing nutrient absorption, and supporting overall growth and development (Bravi et al. 2016). Lactoferrin, for instance, possesses antimicrobial properties and aids in iron absorption, whereas growth factors stimulate tissue repair and cellular growth (Woodman et al. 2018). Colostrum also acts as a natural probiotic, fostering the growth of beneficial gut bacteria and establishing a healthy gut microbiota in the newborn. Human milk oligosaccharides (HMOs) present in colostrum serve as prebiotics, promoting the growth of beneficial bacteria like *Bifidobacteria*, which play a crucial role in gut health and immune system development (Okburan and Kızıler 2023; Berger et al. 2020).

**TABLE 1:** Chemical composition of human colostrum.

Nutritional factor (per 100 mL of colostrum)	Human colostrum
Energy (kcal)	58
Protein (g)	3.7
Lactose (g)	5.3
Fat (g)	2.9
*Immune factors (mg/mL)*	
Lactoferrin	700
IgA	17.35
IgG	0.43
*Growth factors*	
Insulin‐like growth factor (IGF)	18 mg/L
Growth hormone (GH)	41 ng/L
Epidermal growth factor (EGF)	200mcg/L

*Source:* Godhia and Patel (2013).

The transition from colostrum to mature milk occurs over the first few days to weeks postpartum (Pandey et al. 2021; Berger et al. 2020). As lactation progresses, there is a gradual increase in milk volume, accompanied by changes in milk composition (Pandey et al. 2021). The protein and antibody content decreases, whereas the fat and lactose content increases, reflecting the changing nutritional needs of the growing infant (Verduci, Giannì, and Benedetto 2020; Godhia and Patel 2013). The primary composition of human mature breast milk consists of 87% water, 3.8% fat, 1.0% protein, and 7% lactose (Pandey et al. 2021; Berger et al. 2020; Verduci, Mameli, et al. 2020). Fat and lactose serve as the primary sources of energy essential for the growing infant (Miolski et al. 2022). Notably, the lipid fraction of breast milk contains essential fatty acids, fat‐soluble vitamins, and long‐chain polyunsaturated fatty acids crucial for neurological and visual development (Bahreynian et al. 2020). Linoleic acid and alpha‐linolenic acid act as precursors to long‐chain polyunsaturated fatty acids, such as arachidonic acid and docosahexaenoic acid. These long‐chain polyunsaturated fatty acids are vital for the development of the infant's brain and retina (Lapillonne and Moltu 2016).

Breastfeeding has been associated with beneficial effects on gut health and the establishment of a healthy gut microbiota in infants. Human breast milk contains prebiotics, probiotics, and antimicrobial factors that promote the growth of beneficial bacteria, such as *Bifidobacteria* and *Lactobacilli*, while inhibiting the growth of pathogenic bacteria (Pandey et al. 2021; Okburan and Kızıler 2023). These beneficial bacteria play a crucial role in immune function, nutrient absorption, and overall gut health. Furthermore, breast milk is rich in bioactive components like HMOs (Okburan and Kızıler 2023; Berger et al. 2020). Human milk oligosaccharides play a pivotal role in modulating the infant gut microbiota and providing protection against pathogens (Berger et al. 2020).

Lactose stands as the predominant carbohydrate in human milk, playing an indispensable role in fostering the growth and development of infants (Bravi et al. 2016; Pandey et al. 2021; Berger et al. 2020). Serving as an easily digestible and readily available source of energy, lactose meets the high metabolic demands of infants during their crucial early stages of life (Miolski et al. 2022; Cheema et al. 2021). The brain, being one of the most energy‐intensive organs during infancy, relies heavily on lactose as its primary energy source (Cheema et al. 2021). This promotes optimal cognitive development and supports neurological functions essential for early learning and growth. Beyond energy provision, lactose facilitates the absorption of vital minerals like calcium and phosphorus (Lind et al. 2018). These minerals are pivotal for bone mineralization, ensuring robust skeletal development and overall physical growth in infants. This absorption lays a solid groundwork for future skeletal health, ensuring the formation of strong bones and teeth (Miolski et al. 2022). Furthermore, lactose acts as a natural prebiotic, fostering the growth of beneficial bacteria, notably *Bifidobacteria*, within the infant's gut (Pandey et al. 2021; Okburan and Kızıler 2023). A thriving gut microbiome is crucial for digestive health, immune system modulation, and overall well‐being. By bolstering the presence of beneficial gut flora, lactose fortifies a protective shield against harmful pathogens, thereby mitigating the risk of gastrointestinal infections and associated ailments (Cheema et al. 2021; Lind et al. 2018). Moreover, lactose contributes to the maturation and optimal functioning of the infant's immune system. It aids in the production of sialic acid, a compound vital for the development and activity of specific immune cells (Berger et al. 2020). This, in turn, amplifies the infant's immune response, enhancing their resilience against various infections and diseases (Pandey et al. 2021; Berger et al. 2020).

Lactose intolerance in human breast milk is a rare condition that can occur due to various factors affecting lactose synthesis or secretion (Heine et al. 2017). Unlike the lactose intolerance commonly seen in older children and adults, where there is a deficiency of the enzyme lactase needed to digest lactose, lactose intolerance in breast milk is typically not due to the presence of lactose but rather to other components or conditions (Costanzo and Canani 2018). One possible cause of lactose intolerance in breast milk is maternal diet. Consuming certain foods or medications can alter the composition of breast milk, potentially leading to symptoms of lactose intolerance in the infant (Bravi et al. 2016). Additionally, conditions affecting the mother's ability to produce or secrete milk components, such as hormonal imbalances or breast infections, can also contribute to lactose intolerance in breastfed infants (Heine et al. 2017; Costanzo and Canani 2018). It is essential to differentiate between true lactose intolerance and other conditions that may mimic its symptoms, such as cow's milk protein intolerance or gastrointestinal infections. Accurate diagnosis is crucial to ensure appropriate management and treatment for affected infants (Darma et al. 2024).

Human milk is a nutritionally wholesome source, brimming with a diverse array of proteins, including casein and whey proteins, growth factors, enzymes, and hormones that are vital for infant growth and development (Cheema et al. 2021; Abdullayeva and Abdullaeva 2021). Among its protein components, casein and whey proteins stand out, with casein dominating the protein fraction while whey proteins, including lactoferrin, immunoglobulins, α‐lactalbumin, and serum albumin, offer bioactive functions crucial for immune support and nutrient delivery (Bzikowska‐Jura et al. 2018). Lactoferrin, for instance, showcases antimicrobial properties and aids in iron absorption, bolstering the infant's immune system against infections (Czosnykowska‐Łukacka et al. 2019). Meanwhile, lactalbumin not only provides essential amino acids for growth but also possesses antimicrobial and immunomodulatory effects, safeguarding against pathogens (Garcia‐Rodenas et al. 2019). Serum albumin, though present in smaller amounts, plays a key role as a carrier protein, transporting vital substances like fatty acids and hormones throughout the bloodstream. Its contributions extend to maintaining fluid balance and aiding nutrient absorption in the infant's body (Garcia‐Rodenas et al. 2019; Vass et al. 2023).

Moreover, human milk consists of growth factors, enzymes, and hormones that coordinate critical processes in infant development. The complex interplay of these components underscores the dynamic and personalized nature of breast milk, making it an unparalleled and invaluable source of nutrition for infants. Growth factors like epidermal growth factor (EGF), insulin‐like growth factor (IGF), and transforming growth factor‐beta (TGF‐β) drive the growth and maturation of organs, such as the intestines and immune system, ensuring the infant thrives (Gila‐Diaz et al. 2019). Enzymes such as lipase, amylase, and lactase assist in the digestion and absorption of nutrients, whereas hormones like leptin and ghrelin regulate appetite, metabolism, and growth (Gila‐Diaz et al. 2019; Bzikowska‐Jura et al. 2018). The presence of digestive enzymes in breast milk, such as lipases and proteases, aids in the efficient digestion and absorption of nutrients, promoting gut health and reducing the risk of gastrointestinal issues in infants (Hernell and Lonnerdal 2019).

Breast milk is easily digestible and is designed for the infant's digestive capabilities, containing the optimal ratio of whey to casein proteins (Bzikowska‐Jura et al. 2018; Abdullayeva and Abdullaeva 2021). In breast milk, whey and casein are the primary proteins present, with whey being easier for infants to digest than casein (Bzikowska‐Jura et al. 2018). The ratio of whey to casein in breast milk is not fixed; it varies throughout the feeding process and as the infant grows (Abdullayeva and Abdullaeva 2021). Casein contains bioactive peptides that may have various physiological functions, including immune modulation and blood pressure regulation (Pandey et al. 2021; Berger et al. 2020; Abdullayeva and Abdullaeva 2021). It is a slow‐digesting protein that forms a curd in the stomach, providing a sustained release of amino acids (Lind et al. 2018). This slow digestion can help infants feel full for longer periods, supporting regular feeding patterns and promoting healthy weight gain (Cheema et al. 2021; Abdullayeva and Abdullaeva 2021). Initially, breast milk has a higher whey content, which is quickly digested, providing immediate nourishment to the newborn. This rapid digestion is essential for the newborn's small stomach capacity and high metabolic rate (Cheema et al. 2021). As the feeding progresses, the concentration of casein in breast milk increases. Casein is a slow‐digesting protein that provides longer‐lasting satiety and ensures sustained energy and nutrient supply for the growing infant (Abdullayeva and Abdullaeva 2021). This dynamic shift in protein composition in breast milk is modified to meet the evolving nutritional needs of the infant at different stages of development, supporting optimal growth and overall health (Lind et al. 2018; Abdullayeva and Abdullaeva 2021). The balanced presence of both casein and whey proteins in human milk ensures a harmonious and synergistic nutritional profile (Woodman et al. 2018; Costanzo and Canani 2018; Jung et al. 2016). Although casein provides sustained energy and promotes satiety, whey offers rapid nutrition and immune support.

Nonprotein nitrogen compounds in human breast milk encompass a diverse array of bioactive molecules that play crucial roles in infant nutrition and development. These compounds, constituting approximately 25% of the total nitrogen content, include endogenous peptides, urea, nucleotides, carnitine, creatine, free amino acids, DNA, and RNA (Lyons et al. 2020). Among these compounds, free amino acids stand out as essential nutrients readily available for absorption by the infant without the need for prior digestion. Throughout lactation, the levels of free amino acids exhibit dynamic changes specific to each amino acid, reflecting the infant's evolving nutritional requirements (Lönnerdal et al. 2017). Glutamine, glutamate, glycine, serine, and alanine are among the free amino acids that consistently increase during the first months of lactation, supporting various aspects of infant development, including intestinal barrier function, immune system modulation, and growth (Van Sadelhoff et al. 2020). Taurine, another important nonprotein nitrogen compound abundant in breast milk, plays vital roles in excitable tissues, oxidative stress modulation, and osmoregulation (Lyons et al. 2020). Its presence in breast milk is particularly critical for preterm infants who may have limited endogenous synthesis capacity. Taurine deficiency in artificial formulas has been associated with vision and hearing problems, impaired fat absorption, and liver complications (Cao et al. 2018). Carnitine, derived from amino acids lysine and methionine, facilitates the transport of long‐chain fatty acids and supports their metabolism (Lyons et al. 2020). Human milk provides a rich source of carnitine, aiding in the utilization of fatty acids and exerting neuroprotective effects, especially crucial during infancy when plasma carnitine concentrations decline markedly (Alhasaniah 2023). Polyamines, small metabolites derived from amino acids, play pivotal roles in organ growth, immune system regulation, and intestinal maturation (Lyons et al. 2020). Human milk contains biologically active polyamines, such as putrescine, spermidine, and spermine, which contribute to the development of the gastrointestinal microbiota and immune system in infants (Muñoz‐Esparza et al. 2023).

The milk fat globule membrane is a complex structure found in breast milk that surrounds the fat droplets (Gallier et al. 2015). Composed of various lipids, proteins, and glycoproteins, the milk fat globule membrane plays a crucial role in the health and development of infants (Brink and Lönnerdal 2020). One of the key components of the milk fat globule membrane is phospholipids, such as sphingomyelin and phosphatidylcholine, which are essential for brain development and function (Wilmot et al. 2025). Moreover, sphingomyelin is abundant in the milk fat globule membrane and has been linked to cognitive development and the formation of neural connections in infants (Gallier et al. 2015; Brink and Lönnerdal 2020). Additionally, the milk fat globule membrane contains bioactive proteins and peptides with various functions. For example, lactadherin, also known as MFG‐E8, plays a role in immune function and has anti‐inflammatory properties (Aziz et al. 2017). Another protein found in the milk fat globule membrane, butyrophilin, is involved in immune regulation and may contribute to the development of the infant's immune system (Lee et al. 2018; Brink and Lönnerdal 2020). Furthermore, the milk fat globule membrane contains glycoproteins and glycolipids, which are involved in cell signaling and communication. These molecules help regulate various physiological processes in the infant's body, including immune function, digestion, and metabolism (Gallier et al. 2015).

Milk exosomes, secreted by mammary gland epithelial cells and released from milk fat globules during lactation, are vesicles containing various lipids, proteins, and genetic material, such as microRNA (Liao et al. 2017; Zempleni et al. 2017). This encapsulation affords them protection against digestive degradation, enabling them to be captured by cellular endocytosis and deliver their cargo to recipient cells. The bioactive molecules transported by exosomes have significant effects on immunity, growth and development, cell proliferation, and the differentiation of progenitor cells in lung epithelium (Zempleni et al. 2017). However, the full biological and nutritional importance of the lipids and proteins found in breast milk exosomes remains to be fully understood (Gallier et al. 2015). Although initially characterized in human colostrum and breast milk, exosomes can also be obtained from bovine milk on a significant scale, and they demonstrate cross‐species tolerance (Melnik et al. 2021). This suggests the potential for utilizing exosomes from various sources, including bovine milk, to harness their biological functions and benefits (Ruan et al. 2025). Further research into the composition and functions of milk exosomes holds promise for understanding their role in infant nutrition and development, as well as their potential applications in health and medicine. The discovery and exploration of these tiny vesicles open exciting avenues for enhancing our understanding of milk biology and leveraging its bioactive components for human health (Celik et al. 2024).

Minerals and vitamins are essential micronutrients found in human milk that play critical roles in supporting the growth, development, and overall health of infants (Lind et al. 2018). Casein‐bound calcium is readily absorbed by infants, contributing to bone mineralization and skeletal development (Heine et al. 2017; Jung et al. 2016). Iron in breast milk plays a vital role in supporting the growth and development of infants during the early stages of life. Although the concentration of iron in breast milk is relatively low compared with formula milk or fortified foods, it is highly bioavailable, meaning that a significant proportion of the iron in breast milk can be easily absorbed and utilized by the infant's body (Cai et al. 2015). Despite the relatively low iron content, exclusive breastfeeding is recommended for the first 6 months of life by organizations like the World Health Organization (WHO 2021). This is because the bioavailable iron in breast milk, combined with the infant's own iron stores accumulated during the last trimester of pregnancy, is generally sufficient to meet the baby's iron needs during this period. Vitamin A in breast milk is crucial for supporting the growth, development, and overall health of infants during the early stages of life. Breast milk is a natural and rich source of bioavailable vitamin A, providing infants with the essential retinol and carotenoids they need for optimal growth and development (Lind et al. 2018). Iodine is an essential micronutrient present in breast milk, playing a crucial role in supporting the growth and development of infants, particularly in the early stages of life. Adequate iodine intake is essential for the synthesis of thyroid hormones, which are critical for normal growth, metabolism, and cognitive development in infants and children (Pandey et al. 2021; Cai et al. 2015).

Foremilk and hindmilk are terms commonly used to describe the two different types of breast milk produced during a feeding session, each with its own unique composition and benefits for the infant (Pandey et al. 2021; Takumi et al. 2022). Understanding the distinction between these two types of milk is crucial for ensuring optimal nutrition and growth for the baby. Foremilk, also known as “pre‐mature milk,” is the milk initially released at the beginning of a feeding session. It is typically lower in fat content and has a higher water content than hindmilk (Ojo‐Okunola et al. 2020). Foremilk plays an important role in hydrating the infant and quenching their thirst due to its higher water content (Takumi et al. 2022). However, excessive consumption of foremilk without adequate intake of hindmilk can lead to issues, such as colic, gas, and frequent, frothy stools due to its high lactose content (Takumi et al. 2022; Ojo‐Okunola et al. 2020).

On the contrary, hindmilk, also referred to as “mature milk,” is the milk that is released toward the end of a feeding session. It is richer in fat and provides essential nutrients and calories needed for the baby's growth and development (Pandey et al. 2021; Van Sadelhoff et al. 2020). Hindmilk has a creamier consistency due to its higher fat content, which helps to satisfy the baby's hunger and promote weight gain (Takumi et al. 2022). Adequate consumption of hindmilk is essential for ensuring proper growth, development, and overall well‐being of the infant (Van Sadelhoff et al. 2020; Ojo‐Okunola et al. 2020). During a breastfeeding session, the transition from foremilk to hindmilk occurs gradually as the baby continues to nurse and empty the breast (Pandey et al. 2021). It is important for mothers to allow the baby to feed on one breast until it is fully drained before offering the other breast to ensure that the infant receives a balanced intake of both foremilk and hindmilk (Takumi et al. 2022; Ojo‐Okunola et al. 2020). This approach helps to ensure optimal nutrition and avoids potential issues associated with imbalanced milk consumption.

Table 2 presents a comparative analysis of the composition of human milk alongside other mammalian milks, such as cow, goat, buffalo, and camel. The distinct composition of breast milk underscores species‐specific adaptations aimed at fulfilling the unique nutritional requirements of offspring. A notable distinction lies in the protein content. Human breast milk contains a lower protein concentration than cow's milk, which boasts a higher protein content (Zhang et al. 2016; Pietrzak‐Fiećko and Kamelska‐Sadowska 2020). This variance is indicative of the differing growth rates and metabolic needs between human infants and calves. Moreover, the lipid profile of human milk stands apart from that of other mammalian milks. Human milk is characterized by elevated levels of unsaturated fatty acids and cholesterol, which are vital for neural and vascular development (Bahreynian et al. 2020). Carbohydrate composition is another area of divergence among mammalian milks. Human milk is predominantly composed of lactose, whereas other mammalian milks may feature different types of sugars (Silanikove et al. 2015). Additionally, human breast milk is enriched with unique bioactive compounds, such as HMOs and growth factors. These bioactive components offer added advantages for infant health and development, further distinguishing human breast milk from other mammalian milks (Backhed et al. 2015; Berger et al. 2020).

**TABLE 2:** Comparative composition of human milk and other mammalian milks.

Nutrient/component	Human milk	Cow's milk	Goat's milk	Buffalo's milk	Camel's milk
Water	87%	87%	87%	82%	87%
Fat	3.8%	3.4%	4.1%	8%	3.4%
Protein	1%	3.2%	3.6%	4.5%	3.1%
Lactulose	7%	4.8%	4.5%	4.7%	4.4%
Omega 3 fatty acids	High	Low	Moderate	High	High
Omega 6 fatty acids	Moderate	High	Moderate	Moderate	Moderate
Immunoglobulins (Ig A)	Present	Present	Present	Present	Present
Growth factors	Present	Present	Present	Present	Present
Human milk oligosaccharides	Present	Absent	Absent	Absent	Absent

*Source:* Silanikove et al. (2015), Zhang et al. (2016), Pietrzak‐Fiećko and Kamelska‐Sadowska (2020).

## 4 Health and Nutritional Related Benefits of Breastfeeding for Infants

Breastfeeding is universally acknowledged as the gold standard for infant nutrition, offering an array of both short‐term and long‐term health advantages crucial for an infant's overall health. In addition to supplying essential nutrients and antibodies vital for the baby's immune system, breastfeeding also nurtures a unique bond between the mother and child. Numerous studies and health organizations, including the WHO and the American Academy of Pediatrics (AAP), advocate for exclusive breastfeeding for the first 6 months of an infant's life, highlighting its unmatched importance in fostering optimal infant health (Balogun et al. 2015). Breastfeeding after 6 months of age continues to provide numerous health benefits for both infants and mothers. Although introducing complementary foods around this time is common, the WHO recommends continued breastfeeding alongside appropriate complementary foods for up to 2 years or beyond (Balogun et al. 2015; Zielinska et al. 2017).

## 5 Short‐Term Health and Nutritional Benefits of Breastfeeding for Infants

Breastfeeding imparts a variety of short‐term health benefits to infants, encompassing protection against infections, management of infantile colic, enhancement of immune system development, optimal nutritional balance, and reinforcement of the mother–child bond (Table 3). Breast milk's unique composition, coupled with the emotional connection fostered during breastfeeding, significantly contributes to infants' health and well‐being during their early life stages. Encouraging breastfeeding initiation and supporting breastfeeding mothers are crucial steps to amplify these short‐term health benefits, ensuring a healthier beginning for the infant.

**TABLE 3:** Short‐term health and nutritional benefits of breastfeeding for infants.

Health and nutritional benefit	Protective effect of breast milk	References
Comprehensive and balanced nutrition source	Supplies all essential nutrients required for healthy growth and development of infants	Pandey et al. (2021)
Prevention of malnutrition and micronutrient deficiencies	Composition of breast milk adjusts to meet the changing nutritional needs of the growing infant and guards against malnutrition and micronutrient deficiencies, particularly in resource‐limited settings	Brahm and Valdes (2017), Bakar et al. (2018), Gomez‐Acebo et al. (2021)
Strengthening the infant's immune system	Breast milk contains various immune cells, including lymphocytes, macrophages, and neutrophils, which prime the infant's immune system and enhance its ability to fight infections	Backhed et al. (2015)
Immune system maturation	The unique composition of breast milk, comprising cytokines, growth factors, and other bioactive components, supports the maturation of the infant's immune system	Bzikowska‐Jura et al. (2018), Gila‐Diaz et al. (2019)
Reduced frequency and severity of diarrhea (e.g., rotavirus diarrhea)	Breastfeeding significantly diminishes both the frequency and severity of diarrhea episodes in infants, attributed to the rich content of antibodies and protective factors in breast milk	Lamberti et al. (2011), Oktaria et al. (2017)
Protection against gastrointestinal infections	Breast milk serves as a natural defense mechanism against gastrointestinal infections, reducing the risk of conditions like sepsis and necrotizing enterocolitis in infants	Sisk et al. (2017), Cortez et al. (2018)
Passive immunity	Colostrum and mature breast milk contain antibodies, white blood cells, and other immune components that provide passive immunity to newborns, protecting them from various pathogens	Palmeira and Carneiro‐Sampaio (2016)
Reduced risk of allergic diseases	Breastfeeding has been associated with diminished rates of allergic diseases, asthma, and eczema during infancy, highlighting its beneficial impact on immune‐mediated conditions	Oddy (2017)
Potential protection against SARS‐CoV‐2	Preliminary research suggests that breast milk from vaccinated mothers may contain antibodies that offer some level of protection against SARS‐CoV‐2 infection in infants, highlighting the multifaceted protective benefits of breastfeeding against viral pathogens	Centeno‐Tablante et al. (2021)
Reduction of infantile colic risk	Breast milk is easily digestible and provides soothing comfort to the infant through skin‐to‐skin contact during breastfeeding	Vandenplas et al. (2016), Hjern et al. (2020)
Alleviation of colic symptoms	Breast milk contains bioactive components, such as probiotics and human milk oligosaccharides, promoting a healthy gut microbiota	Hjern et al. (2020), Daelemans et al. (2018)
Reduction of sudden infant death syndrome	Breast milk contains antibodies, cytokines, and other immune factors that strengthen the infant's immune system, offering protection against infections and inflammation. Breastfeeding involves skin‐to‐skin contact, which helps in stabilizing the infant's physiological regulation	Kinney et al. (2018), Goldberg et al. (2018), Jullien (2021), Alm et al. (2016), Thompson et al. (2017), Dueñas‐Espín et al. (2021), Bartick et al. (2020), Landa‐Rivera et al. (2022)
Prevent the symptoms of colic from becoming more severe or intense	Optimal breastfeeding techniques, including ensuring adequate satiation from one breast before offering the other, help minimize gas‐related pain	Aktas and Alemdar (2019); Ellwood et al. (2020)
Emotional and psychological well‐being of mother and infant	Facilitates emotional attachment and bonding through intimate interactions during breastfeeding	Bigelow and Power (2020), Dueñas‐Espín et al. (2021)
Release of bonding hormones, such as oxytocin	Strengthens the bond between mother and infant, promoting affection, trust, and connection	Uvnas‐Moberg et al. (2019), Brown (2017), Phillipps et al. (2020)
Responsive caregiving	Allows mothers to promptly respond to infant's cues, promoting emotional regulation and stability	Potgieter (2017)

### 5.1 Immune System Development and Protection Against Infections

Breastfeeding is crucial for shaping and strengthening the infant's immune system, laying the groundwork for enduring health benefits. Breast milk encompasses various immune cells, such as lymphocytes, macrophages, and neutrophils, which prime the infant's immune system and bolster its capacity to combat infections (Backhed et al. 2015). Additionally, the unique composition of breast milk, including cytokines, growth factors, and other bioactive components, supports immune system maturation and regulates inflammation in the infant's body (Bzikowska‐Jura et al. 2018; Gila‐Diaz et al. 2019). Moreover, breastfeeding has further been linked to diminished rates of allergic diseases, asthma, and eczema during infancy, highlighting its beneficial impact on immune‐mediated conditions (Oddy 2017).

Breastfeeding serves as a critical shield against infections in infants, backed by an extensive body of scientific evidence. Numerous studies have consistently demonstrated that breastfeeding significantly diminishes both the frequency and severity of diarrhea episodes in infants (Oktaria et al. 2017). The rich content of antibodies and protective factors in breast milk functions as a potent natural defense mechanism against gastrointestinal infections, translating to reduced occurrences of diarrhea (Lamberti et al. 2011). Although breastfeeding has been shown to delay the onset of rotavirus diarrhea, its protective efficacy tends to wane in the second year of life (Webb and Cabada 2018). Breastfeeding has been shown to reduce the risk of sepsis in infants due to the presence of beneficial antibodies and immune‐boosting factors in breast milk (Cortez et al. 2018). Colostrum and mature breast milk contain antibodies, white blood cells, and other immune components that help protect newborns from infections, including sepsis (Palmeira and Carneiro‐Sampaio 2016). The immunological benefits provided by breastfeeding play a crucial role in enhancing the infant's immune response and reducing susceptibility to severe infections like sepsis during the vulnerable neonatal period (Verhasselt 2024). Additionally, breastfeeding has been shown to significantly reduce the risk of necrotizing enterocolitis, a severe gastrointestinal condition commonly seen in premature infants. Given the protective effects of breast milk against necrotizing enterocolitis, breastfeeding may reduce the need for surgical interventions, antibiotic treatments, and hospitalizations related to necrotizing enterocolitis (Cortez et al. 2018; Sisk et al. 2017).

Furthermore, emerging research has illuminated the potential protective role of breastfeeding against severe acute respiratory syndrome coronavirus 2 (SARS‐CoV‐2), the virus responsible for the COVID‐19 pandemic. Preliminary studies suggest that breast milk from vaccinated mothers contains antibodies that may provide some level of protection against SARS‐CoV‐2 infection in infants (Centeno‐Tablante et al. 2021). Although continued investigation is warranted to elucidate the extent and duration of this protection, these findings underscore the multifaceted protective benefits of breastfeeding against various infections, including viral pathogens like SARS‐CoV‐2 (Guo et al. 2025).

### 5.2 Reduction of Sudden Infant Death Syndrome

Sudden infant death syndrome remains a devastating mystery in pediatrics, with its causes and prevention strategies being subjects of intensive research (Kinney et al. 2018). Over the years, various factors have been identified that contribute to the risk of sudden infant death syndrome, and among these, breastfeeding has emerged as a potentially protective factor (Goldberg et al. 2018). Breast milk contains a plethora of bioactive compounds that play a crucial role in immune system development, gut microbiota modulation, and overall infant well‐being (Goldberg et al. 2018). These unique components of breast milk might contribute to its protective role against sudden infant death syndrome.

Several studies have explored the relationship between breastfeeding and sudden infant death syndrome risk. A comprehensive review by Jullien (2021) provided substantial evidence supporting the protective effect of breastfeeding against sudden infant death syndrome. Several research studies indicated that any breastfeeding, even if not exclusive, was associated with a reduction in sudden infant death syndrome risk (Alm et al. 2016). Furthermore, exclusive breastfeeding showed an even stronger protective effect against sudden infant death syndrome, underscoring the importance of exclusive breastfeeding in infant health outcomes (Landa‐Rivera et al. 2022).

A more recent study by Thompson et al. (2017) delved deeper into the duration of breastfeeding and its association with sudden infant death syndrome risk. This study found that breastfeeding for at least 2 months was associated with a significant reduction in the risk of sudden infant death syndrome. Notably, the protective effect of breastfeeding against sudden infant death syndrome increased with longer durations of breastfeeding. Specifically, infants breastfed for 2–4 months had a 40% reduced risk of sudden infant death syndrome, whereas those breastfed for more than 6 months had a 64% reduced risk compared with those not breastfed or breastfed for less than 2 months. The mechanisms through which breastfeeding might reduce the risk of sudden infant death syndrome are multifactorial. One proposed mechanism is the immunological benefits of breast milk. Breast milk contains antibodies, cytokines, and other immune factors that bolster the infant's immune system, offering protection against infections and inflammation (Thompson et al. 2017; Lokossou et al. 2022). Given that some sudden infant death syndrome cases have been associated with infections, the immune‐boosting properties of breast milk might play a role in reducing sudden infant death syndrome risk (Kinney et al. 2018).

Additionally, the act of breastfeeding itself promotes better physiological regulation in infants. Breastfeeding involves skin‐to‐skin contact, which helps in stabilizing the infant's heart rate, temperature, and respiratory patterns (Dueñas‐Espín et al. 2021). These physiological benefits of breastfeeding might contribute to reduced sudden infant death syndrome risk by enhancing infant autonomic regulation (Bartick et al. 2020). Although the evidence supporting the protective role of breastfeeding against sudden infant death syndrome is robust, it is essential to recognize that breastfeeding is just one of the many factors influencing sudden infant death syndrome risk. Other factors, such as infant sleeping position, sleep environment, and maternal smoking, also play significant roles in sudden infant death syndrome risk (Kinney et al. 2018).

### 5.3 Nutritional Adequacy

Breast milk serves as a comprehensive and balanced nutrition source for infants, supplying all essential nutrients required for healthy growth and development (Pandey et al. 2021). The composition of breast milk adapts to meet the evolving nutritional demands of the growing infant, ensuring optimal nutrient absorption and utilization by the infant's body (Brahm and Valdes 2017). Furthermore, breastfeeding guards against malnutrition and micronutrient deficiencies, especially in resource‐limited settings where access to safe and nutritious food might be restricted (Bakar et al. 2018; Gomez‐Acebo et al. 2021). The WHO underscores the critical role of breastfeeding in preventing malnutrition, emphasizing its unparalleled nutritional value and its potential to bridge nutritional gaps, particularly in vulnerable populations (WHO 2016). Breastfeeding not only provides a rich array of nutrients but also offers bioactive components and protective factors that support overall health and well‐being, thereby serving as a remarkable supporter against malnutrition and its detrimental consequences (Modak et al. 2023).

### 5.4 Management of Infantile Colic

Infantile colic is classified as a functional gastrointestinal disorder, and its exact cause remains elusive (Indrio et al. 2015). However, factors such as an imbalanced gastrointestinal microbiome, increased intestinal permeability, chronic inflammation, and behavioral elements like over‐ or under‐stimulation are believed to play a role (Zeevenhooven et al. 2018; Vandenplas et al. 2016). For healthcare professionals, distinguishing between functional and organic causes is challenging, adding to the complexity of diagnosis. Parental stress can exacerbate the situation, creating a vicious cycle as babies tend to cry more, further increasing parental stress levels. To this end, several studies have highlighted the potential benefits of breastfeeding in reducing both the risk and severity of infantile colic (Vandenplas et al. 2016). Breast milk is easily digestible, and the skin‐to‐skin contact during breastfeeding can provide soothing comfort to the infant. Furthermore, breast milk contains bioactive components, such as probiotics and HMOs, which promote a healthy gut microbiota and may help alleviate colic symptoms (Hjern et al. 2020). The manipulation of the gastrointestinal microbiome through probiotic supplementation has also shown promise. Specifically, *Lactobacillus reuteri* DSM 19378 is the most extensively studied probiotic for colic, particularly in breastfed infants (Daelemans et al. 2018). Although breastfeeding is considered a primary protective factor against colic, certain breastfeeding practices may inadvertently exacerbate symptoms. For instance, attempting to breastfeed from both breasts during a single feeding session can lead to excessive intake of lactose‐rich foremilk, causing gas and discomfort in infants (Aktas and Alemdar 2019). Optimal breastfeeding technique is crucial; the infant should be breastfed until adequately satiated from the first breast before offering the second. Poor breastfeeding techniques, such as improper positioning and latch, can cause babies to swallow air or experience gas‐related pain (Aktas and Alemdar 2019; Ellwood et al. 2020). Interestingly, concerns over perceived insufficient milk supply and the anxiety stemming from frequent crying episodes are significant factors influencing mothers to consider formula feeding as an alternative (Aktas and Alemdar 2019; Sorkhabi et al. 2020). This highlights the need for comprehensive support and education for breastfeeding mothers to ensure proper technique and to address concerns related to milk supply and infant‐feeding behaviors. Although breastfeeding offers numerous benefits in reducing the risk and severity of infantile colic, it is essential to educate and support mothers in optimal breastfeeding practices to maximize these benefits and alleviate concerns related to milk supply and infant‐feeding (Purkiewicz et al. 2025).

### 5.5 Bonding and Emotional Well‐Being

Breastfeeding serves as a cornerstone for nurturing the emotional and psychological well‐being of both mother and infant, fostering a deep and profound bond through intimate interactions, such as skin‐to‐skin contact, eye contact, and physical closeness (Bigelow and Power 2020). These intimate moments during breastfeeding are not merely acts of nourishment but also powerful mechanisms that facilitate emotional attachment, promoting a sense of security and trust between the mother and child (Dueñas‐Espín et al. 2021).

The physiological process of breastfeeding is further enriched by the release of bonding hormones, primarily oxytocin, often referred to as the “love hormone” or “bonding hormone.” Oxytocin plays a focal role in strengthening the bond between mother and infant by enhancing feelings of affection, trust, and connection (Uvnas‐Moberg et al. 2019). This hormonal surge during breastfeeding not only facilitates maternal–infant bonding but also contributes to maternal well‐being, reducing stress levels and promoting feelings of relaxation and contentment (Brown 2017; Innis 2014).

Moreover, breastfeeding provides a unique avenue for mothers to respond promptly to their infant's cues and needs, promoting responsive and nurturing caregiving. This responsive caregiving associated with breastfeeding plays a crucial role in emotional regulation for the infant, helping them navigate their emotional world more effectively and promoting a sense of security and stability (Potgieter 2017). In essence, breastfeeding transcends its primary role as a nutritional source to become a powerful catalyst for emotional bonding and well‐being, laying a solid foundation for healthy psychosocial development and nurturing lifelong emotional resilience.

## 6 Long‐Term Nutritional and Health Benefits of Breastfeeding for Infants

Breastfeeding is known to provide numerous short‐term nutritional and health benefits for infants, but its advantages extend well beyond infancy, contributing to the long‐term health and well‐being of children into later stages of life. Table 4 explores the evidence supporting the long‐term nutritional and health benefits of breastfeeding for babies as they progress through childhood and into adulthood, encompassing aspects, such as reduced risk of chronic diseases, cognitive development, and psychosocial outcomes.

**TABLE 4:** Long‐term health and nutritional benefits of breastfeeding for infants.

Health and nutritional benefit	Protective effect of breast milk	References
Reduced risk of obesity and type 2 diabetes in childhood and adulthood	Breastfeeding provides optimal nutrition and helps regulate infant appetite, contributing to healthier growth and weight management	Bjerregaard et al. (2019), Fields et al. (2016), Yasmeen et al. (2019), Horta and de Lima (2019), Dewey et al. (2021a), Mamun et al. (2015)
Reduced risk of type 1 diabetes in children	Breast milk contains immunomodulatory components that support the development of the infant's immune system, potentially reducing the risk of autoimmune reactions leading to type 1 diabetes	Nolan et al. (2020), Hakola et al. (2018)
Reduced risk of cardiovascular disease in adulthood	Breastfed infants have lower blood pressure and improved cholesterol profiles in adulthood, likely mediated through breastfeeding's effects on metabolic health, endothelial function, and inflammation	Horta et al. (2015b), Tschiderer et al. (2022), Rak et al. (2016), Choi et al. (2017)
Protective effect against childhood leukemia	Breastfeeding, especially when initiated early and continued for longer durations, is associated with a reduced risk of childhood leukemia, possibly through immunological and gut microbiota modulation	Amitay et al. (2016), Amitay and Keinan‐Boker (2015), Su et al. (2021), Whitehead et al. (2016), Ogg et al. (2020), Fox et al. (2015), Davis et al. (2022), Bai et al. (2018), Furci et al. (2023)
Potential protective effects against osteoarthritis	Breastfeeding may confer protective effects against osteoarthritis through early‐life metabolic programming, optimal bone development, and joint health.	Haschke et al. (2016), Palmer et al. (2018), Wood et al. (2015), Saavedra (2022)
Reduced risk of otitis media (middle ear infection)	Breastfeeding is associated with a reduced risk of otitis media during infancy and early childhood, with exclusive breastfeeding for at least 6 months offering protection against recurrent otitis media in the first 2 years of life	Quigley et al. (2016), Kørvel‐Hanquist et al. (2017), Brennan‐Jones et al. (2017), Bowatte et al. (2015)
Protection against respiratory infections	Breastfeeding provides protection against various respiratory infections, including pneumonia, bronchiolitis, and upper respiratory tract infections, by promoting the development of a robust immune system and supporting optimal lung development	Pandolfi et al. (2019), Frank et al. (2019), Tromp et al. (2017), Hanson and Winberg (2016), Hakansson et al. (2018)
Protection against gastrointestinal infections	Breastfeeding offers protection against gastrointestinal infections, such as diarrhea and gastroenteritis by establishing and maintaining a healthy gut microbiota through antimicrobial factors, prebiotics, and probiotics present in breast milk	Frank et al. (2019), Carr et al. (2021), Ajetunmobi et al. (2015), Payne and Quigley (2016), Domenici and Vierucci (2022)
Reduction of dental caries (tooth decay)	Breastfeeding reduces the risk of early childhood caries by maintaining a balanced oral microbiome and inhibiting the proliferation of cariogenic bacteria present in the mouth	Peres et al. (2018), Cui et al. (2017), Brahm and Valdes (2017), Al‐Shehri et al. (2015)
Prevention of malocclusion (misalignment of teeth)	Prolonged breastfeeding is associated with a lower prevalence of malocclusion in children, likely due to the sucking mechanism involved in breastfeeding promoting proper jaw development and muscle coordination	Doğramacı et al. (2016), Peres et al. (2015)
Protection against periodontal diseases (gingivitis and periodontitis)	Breastfeeding may have protective effects against periodontal diseases in adulthood, potentially attributed to the anti‐inflammatory and immunomodulatory components of breast milk promoting oral health and tissue repair	Wang et al. (2023), Heo and Lee (2018), Tham et al. (2015), Peres et al. (2018)
Reduction of oral candidiasis (thrush)	Breastfeeding reduces the risk of oral candidiasis in infants by providing antimicrobial factors that inhibit the growth of *Candida* species and maintain a healthy oral flora	Douglas (2021), Branger et al. (2019), Oba et al. (2020), Jaiswal and Kumar (2023)
Shaping the infant's gut microbiota and long‐term implications for disease risk	Breast milk supports the establishment of a diverse and stable gut microbiota in infants, characterized by higher concentrations of beneficial bacteria, such as Bifidobacterium and Lactobacillus, promoting gut health. Breastfeeding during infancy is associated with a reduced risk of gastrointestinal disorders, such as irritable bowel syndrome, celiac disease, and inflammatory bowel disease, in childhood and adulthood. Early‐life gut microbiota composition may influence the risk of chronic diseases, such as obesity, diabetes, and neurodevelopmental disorders in later life	Kamphorst et al. (2021), Torun et al. (2021), Bertin et al. (2023), Sarkar et al. (2021), Daliry and Pereira (2021), Van den Elsen et al. (2019), Ma et al. (2020), Fabiano et al. (2021)
Modulation of the infant's immune system	Breastfeeding provides bioactive components that modulate the infant's immune system, promoting the growth of beneficial bacteria while inhibiting pathogenic microbes, reducing the risk of gastrointestinal infections	Backhed et al. (2015), Berger et al. (2020), Ma et al. (2020)
Support of gut barrier function	Breast milk promotes mucin production, enhances tight junction integrity, and regulates antimicrobial peptide secretion, supporting gut barrier function and reducing the risk of gastrointestinal disorders, autoimmune diseases, and systemic inflammation	Xie et al. (2022), Walker and Iyengar (2015), Figueroa‐Lozano and Vos (2019)
Promotion of optimal brain growth and cognitive function	Breast milk provides essential nutrients, particularly long‐chain polyunsaturated fatty acids, critical for brain development and function, leading to improved neurodevelopmental outcomes, cognitive performance, language development, and academic achievement in children and adolescents	De‐Weerth et al. (2023), Berger et al. (2020), Pandey et al. (2021), Carlson and Colombo (2016), Basak et al. (2021), Anderson, Vaillancourt, et al. (2016), Binns and Lee (2019), Mohammed et al. (2022)
Long‐term cognitive benefits extending into adulthood	Breastfeeding duration and exclusivity during infancy are associated with higher intelligence quotient scores, better academic performance, increased educational attainment, enhanced cognitive stimulation, socioemotional support, and neurodevelopmental advantages, contributing to success in school and future endeavors in adulthood	Victora et al. (2015), Sajjad et al. (2015), Mohammed et al. (2022), Muktamath et al. (2023)
Potential protective effects against multiple sclerosis	Multiple sclerosis is indeed an autoimmune disease affecting the central nervous system, characterized by chronic inflammation, demyelination, gliosis, and neuronal loss. Studies have suggested that breastfeeding may have a protective effect against the development of multiple sclerosis, particularly in children. The mechanisms underlying this protective effect are not fully understood but may involve the transfer of maternal antibodies and other bioactive components in breast milk, which could modulate the infant's immune system and potentially reduce the risk of autoimmune diseases	Brenton et al. (2017)
Reduction of risk for attention deficit/hyperactivity disorder	Breastfeeding is associated with a reduced risk of Attention deficit/hyperactivity disorder, potentially attributed to essential nutrients, bioactive compounds, bonding, and interaction during breastfeeding supporting optimal brain development, cognitive, and behavioral functions	Gleason and Humphreys (2016), Zeng et al. (2020), Tseng et al. (2019), Victora et al. (2015), Sajjad et al. (2015)
Protection against autism spectrum disorder	Breastfeeding has a protective effect against the risk of autism spectrum disorder, with exclusive breastfeeding showing the lowest risk. This protection acts in a dose‐dependent manner and is thought to be linked to the immunological and neuroprotective properties of breast milk, supporting the infant's immune system and brain development	Ghozy et al. (2020), Husk and Keim (2015), Soke et al. (2019), Martins et al. (2020), Martinat et al. (2021)
Reduced risk of Alzheimer's disease	Breast milk contains essential fatty acids, antioxidants, and growth factors that enhance synaptic connectivity and neural plasticity, potentially reducing neuroinflammation, oxidative stress, and amyloid‐beta accumulation associated with Alzheimer's disease	Carlson and Colombo (2016), Sambra et al. (2021), Chen et al. (2020), Atkinson and Dickman (2023), Andreas et al. (2015), Yadav et al. (2022), Kim et al. (2016), Polverino et al. (2021), Anderson, Vaillancourt, et al. (2016), Binns and Lee (2019), Guzzardi et al. (2020)
Promotion of secure attachment relationships	Breastfeeding fosters a strong bond between mother and baby, facilitating secure attachment relationships through skin‐to‐skin contact, eye contact, and oxytocin release, leading to better emotional regulation, increased maternal responsiveness, and secure attachment behaviors in breastfed infants	Ford et al. (2020), Lawrence and Robert (2021), Innis (2014), Ivanova et al. (2021), Keim et al. (2021)
Enhancement of socioemotional development and behavior	Breastfeeding fosters bonding and attachment, promoting secure and nurturing environments essential for emotional regulation, social skills, and overall well‐being, resulting in better socioemotional outcomes, reduced risk of anxiety, depression, behavioral problems, and enhanced socioemotional development in children and adolescents	Modak et al. (2023), Turner et al. (2019), Rochat et al. (2016), Innis (2014), Ivanova et al. (2021)
Development of effective emotional regulation skills	Breastfeeding provides infants with a comforting and soothing experience, promoting effective emotional regulation skills and stress resilience through close physical contact, rhythmic sucking, and gentle rocking, resulting in reduced cortisol levels, relaxation, better emotional self‐regulation	Uvnas‐Moberg et al. (2019), Hernández‐Vásquez et al. (2022), Modak et al. (2023), Vesel et al. (2015)

### 6.1 Reduced Risk of Noncommunicable Diseases

Breastfeeding has been recognized not only for its immediate health benefits but also for its potential long‐term protective effects against chronic diseases in children. Numerous studies have explored the relationship between breastfeeding and the risk of various chronic noncommunicable disease conditions, revealing compelling evidence in support of breastfeeding's role in reducing the likelihood of developing certain diseases later in life (Capra et al. 2025). One of the most extensively studied long‐term benefits of breastfeeding is its association with a reduced risk of obesity and type 2 diabetes in childhood and adulthood (Bjerregaard et al. 2019). Breastfeeding provides optimal nutrition and helps regulate infant appetite, which may contribute to healthier growth and weight management (Fields et al. 2016; Yasmeen et al. 2019). Several meta‐analyses and cohort studies have demonstrated that breastfed infants are less likely to become overweight or obese compared with formula‐fed infants (Horta and de Lima 2019; Dewey et al. 2021a, 2021b). Furthermore, a longitudinal study published in 2015 found that longer duration of breastfeeding was associated with a lower risk of developing type 2 diabetes in adolescence and adulthood (Mamun et al. 2015). The protective effect of breastfeeding on obesity and diabetes risk may be attributed to the unique composition of breast milk, which includes hormones and bioactive compounds that influence metabolic regulation and fat storage (Fields et al. 2016).

Breastfeeding has been associated with a reduced risk of type 1 diabetes in infants. The immunomodulatory components present in breast milk, such as secretory IgA, lactoferrin, and oligosaccharides, play a vital role in supporting the development and maturation of the infant's immune system, potentially reducing the risk of autoimmune reactions leading to type 1 diabetes (Nolan et al. 2020; Hakola et al. 2018). Moreover, exclusive breastfeeding during the first months of life has been suggested to confer protective effects against the development of type 1 diabetes in genetically susceptible individuals (Nolan et al. 2020).

Breastfeeding has also been linked to a reduced risk of cardiovascular disease later in life. A systematic review and meta‐analysis found that individuals who were breastfed as infants had lower blood pressure and improved cholesterol profiles in adulthood compared with those who were formula‐fed (Horta et al. 2015b). These cardiovascular benefits may be mediated through the long‐term effects of breastfeeding on metabolic health, endothelial function, and inflammation (Tschiderer et al. 2022). Emerging evidence suggests that breastfeeding may also confer protective effects against hypertension in young adults (Rak et al. 2016). Several studies have reported an inverse association between breastfeeding duration and the risk of developing hypertension later in life (Horta et al. 2015b; Choi et al. 2017). The potential mechanisms underlying the protective effects of breastfeeding on hypertension may include improved cardiovascular health, enhanced metabolic regulation, and reduced visceral adiposity, all of which contribute to lower blood pressure levels (Horta et al. 2015b; Tschiderer et al. 2022).

Childhood leukemia encompasses a group of hematological malignancies, with acute lymphoblastic leukemia being the most common subtype, accounting for approximately 75%–80% of cases (Terwilliger and Abdul‐Hay 2017; Zimba 2019). Several epidemiological studies have investigated the association between breastfeeding and the risk of childhood leukemia, yielding intriguing findings that support a protective role for breastfeeding against this malignancy (Amitay et al. 2016). A meta‐analysis conducted by Amitay and Keinan‐Boker (2015) analyzed data from 17 studies and found that 14% to 20% of all childhood leukemia cases may be prevented by breastfeeding for 6 months or more. The protective effect of breastfeeding appears to be dose‐dependent, with longer duration and exclusivity of breastfeeding associated with a greater reduction in leukemia risk (Su et al. 2021). Furthermore, studies have observed a stronger protective effect when breastfeeding is initiated early in infancy, underscoring the importance of breastfeeding as a modifiable risk factor for childhood leukemia (Whitehead et al. 2016; Ogg et al. 2020). The exact mechanisms underlying the protective effect of breastfeeding against childhood leukemia remain speculative, but several hypotheses have been proposed based on the immunological and physiological benefits of breast milk (Amitay et al. 2016). Breast milk contains various bioactive components, such as immunoglobulins, lactoferrin, and cytokines, which play a crucial role in enhancing immune surveillance, modulating inflammation, and promoting apoptosis of potentially cancerous cells (Fox et al. 2015). Furthermore, breastfeeding contributes to the maturation and modulation of the infant's gut microbiota, which plays a pivotal role in immune system development and function (Davis et al. 2022). Disruption of the gut microbiota, termed dysbiosis, has been implicated in the pathogenesis of various diseases, including cancer, suggesting a potential link between breastfeeding, gut microbiota, and leukemia risk (Bai et al. 2018; Furci et al. 2023).

Moreover, breastfeeding has been associated with potential protective effects against osteoarthritis in individuals during later stages of life. Protective mechanisms may involve the provision of essential nutrients and growth factors during breastfeeding, which contribute to optimal bone development and joint health in infancy (Haschke et al. 2016; Palmer et al. 2018). Additionally, breastfeeding's influence on early‐life metabolic programming and body composition may further contribute to reduced osteoarthritis risk in adulthood (Wood et al. 2015; Saavedra 2022). However, further longitudinal studies are needed to confirm these associations and elucidate the underlying mechanisms.

### 6.2 Long‐Term Protection Against Infections in Later Life

Breastfeeding is widely acknowledged for its immediate and long‐term health benefits for infants, providing essential nutrients and antibodies that support their developing immune systems (Quigley et al. 2016). Although the protective effects of breastfeeding against infections during infancy are well established, emerging evidence suggests that breastfeeding may also confer lasting benefits by reducing the risk of infections in later stages of life, including ear infections, respiratory infections, and gastrointestinal infections. Otitis media, commonly known as middle ear infection, is a prevalent childhood illness that can lead to pain, fever, and temporary hearing loss. Several studies have found that breastfeeding is associated with a reduced risk of otitis media during infancy and early childhood (Kørvel‐Hanquist et al. 2017; Brennan‐Jones et al. 2017). A meta‐analysis concluded that exclusive breastfeeding for at least 6 months was protective against recurrent otitis media in the first 2 years of life (Bowatte et al. 2015). The protective effect of breastfeeding on ear infections is believed to be due to the immunological properties of breast milk, including antibodies, cytokines, and other bioactive factors that enhance the infant's immune response and reduce susceptibility to bacterial and viral pathogens (Kørvel‐Hanquist et al. 2017).

Breastfeeding has been shown to provide protection against various respiratory infections, such as pneumonia, bronchiolitis, and upper respiratory tract infections, in both infants and older children (Pandolfi et al. 2019; Frank et al. 2019). The Generation R Study published in 2017 found that breastfeeding duration for 6 months or longer is associated with a reduced risk of lower respiratory tract infections in preschool children (Tromp et al. 2017). The immunomodulatory and anti‐inflammatory components of breast milk play a crucial role in protecting against respiratory infections by promoting the development of a robust and balanced immune system (Frank et al. 2019; Hanson and Winberg 2016). Additionally, breastfeeding provides physical barriers against respiratory pathogens and supports optimal lung development, which may further contribute to reducing the risk of respiratory infections in later life (Hakansson et al. 2018).

Breastfeeding has long been recognized for its protective effects against gastrointestinal infections, including diarrhea and gastroenteritis, which are major causes of morbidity and mortality in children worldwide (Frank et al. 2019). The antimicrobial factors, prebiotics, and probiotics present in breast milk help to establish and maintain a healthy gut microbiota, which is essential for gastrointestinal health and immune function (Carr et al. 2021). Numerous research studies found that exclusively breastfeeding was associated with a reduction in the risk of hospitalization for gastrointestinal infections in early childhood and may reduce morbidity due to infectious illness in infants (Ajetunmobi et al. 2015; Payne and Quigley 2016). The protective effect of breastfeeding on gastrointestinal infections extends into childhood and adolescence, reducing the incidence and severity of diarrheal diseases and other gastrointestinal illnesses (Pandolfi et al. 2019; Frank et al. 2019; Domenici and Vierucci 2022).

### 6.3 Reducing the Risk of Oral Diseases Across the Lifespan

Breastfeeding offers significant long‐term benefits for oral health by reducing the risk of dental caries, malocclusion, periodontal diseases, and oral candidiasis in children and adults (Peres et al. 2018). These protective effects are likely mediated through the immunological, antimicrobial, and nutritional components of breast milk, which support optimal oral development, maintain a balanced oral microbiome, and enhance immune responses against oral pathogens (Tham et al. 2015). Given the lifelong impact of oral diseases on overall health and well‐being, promoting and supporting breastfeeding should be a key public health strategy to improve oral health outcomes across the lifespan. Dental caries, commonly known as tooth decay or cavities, are one of the most prevalent chronic diseases in children worldwide. Breastfeeding has been shown to offer protection against early childhood caries, a specific form of tooth decay that affects infants and toddlers (Cui et al. 2017). The use of baby formulas and bottles has inherent risks because they increase the risk of oral diseases, such as mouth breathing, malocclusion, alteration of bite, and tooth decay (Brahm and Valdes 2017). The natural antibodies and antimicrobial properties present in breast milk help to maintain a balanced oral microbiome, reducing the proliferation of cariogenic bacteria responsible for tooth decay (Al‐Shehri et al. 2015).

Malocclusion refers to misalignment or incorrect positioning of the teeth and jaws, which can lead to various dental and orthodontic problems (Doğramacı et al. 2016). Breastfeeding has been associated with a reduced risk of developing malocclusion and crooked teeth in children. A longitudinal study published in the journal Pediatrics demonstrated that prolonged breastfeeding (more than 12 months) was associated with a lower prevalence of malocclusion in primary and mixed dentition stages (Peres et al. 2015). The sucking mechanism involved in breastfeeding promotes proper jaw development and muscle coordination, which may contribute to the prevention of malocclusion (Doğramacı et al. 2016; Peres et al. 2015).

Periodontal diseases, including gingivitis and periodontitis, are inflammatory conditions affecting the gums and supporting structures of the teeth. Although the direct link between breastfeeding and periodontal health is still an area of ongoing research, some studies suggest that breastfeeding may have protective effects against periodontal diseases later in life (Wang et al. 2023). Research findings found that breastfeeding was associated with a reduced risk of periodontal diseases in adulthood, although the evidence was not conclusive (Heo and Lee 2018). The anti‐inflammatory and immunomodulatory components of breast milk may contribute to maintaining oral health by reducing inflammation and promoting tissue repair in the oral cavity (Peres et al. 2018; Tham et al. 2015).

Oral candidiasis, commonly known as thrush, is a fungal infection of the mouth caused by *Candida* species. Breastfeeding has been shown to reduce the risk of oral candidiasis in infants by providing protective factors that inhibit the growth of *Candida* and maintain a healthy oral flora (Douglas 2021). Research studies demonstrated that exclusive breastfeeding for the first 6 months of life was associated with a lower incidence of oral candidiasis than formula feeding (Branger et al. 2019; Oba et al. 2020). The antimicrobial properties of breast milk, including lactoferrin, lysozyme, and immunoglobulins, help to control the proliferation of *Candida* and other pathogenic microorganisms in the oral cavity (Jaiswal and Kumar 2023).

### 6.4 Nurturing Microflora for Lifelong Well‐Being

Breastfeeding is not just a source of nutrition; it plays a paramount role in shaping the infant's gut microbiota, thereby influencing gut health and overall well‐being (Van den Elsen et al. 2019). The human gut is inhabited by a complex community of microorganisms, collectively known as the gut microbiota, which plays a crucial role in digestion, immune function, and overall health. The composition of an infant's gut microbiota is dynamic and undergoes significant changes during the first few years of life (Gomaa 2020). Breast milk is uniquely tailored to meet the nutritional and immunological needs of the infant and plays a fundamental role in establishing a healthy and diverse gut microbiota (Lyons et al. 2020). Research has shown that breastfed infants have a more diverse and stable gut microbiota than formula‐fed infants, characterized by higher concentrations of beneficial bacteria, such as *Bifidobacterium* and *Lactobacillus* (Ma et al. 2020; Fabiano et al. 2021). These beneficial bacteria play a crucial role in fermenting dietary fibers, producing short‐chain fatty acids, and maintaining gut barrier integrity, thereby contributing to overall gut health (Fabiano et al. 2021). Breast milk contains a myriad of bioactive components, including antibodies, cytokines, growth factors, and antimicrobial peptides, which help to modulate the infant's immune system and protect against infections (Backhed et al. 2015; Berger et al. 2020). These bioactive compounds also play a vital role in shaping the composition and function of the gut microbiota. Studies have demonstrated that breastfeeding promotes the growth of beneficial bacteria while inhibiting the proliferation of pathogenic microbes, thereby enhancing immune function and reducing the risk of gastrointestinal infections (Berger et al. 2020; Ma et al. 2020). The immunomodulatory properties of breast milk contribute to the development of a balanced and robust immune response, which is essential for maintaining gut health and preventing inflammatory conditions, such as inflammatory bowel disease and allergies (Sheikh et al. 2021).

The gut barrier plays a critical role in preventing the translocation of harmful substances, such as toxins and pathogens, from the gut lumen into the bloodstream (Xie et al. 2022). Breastfeeding has been shown to support gut barrier function by promoting the production of mucin, enhancing tight junction integrity, and regulating the secretion of antimicrobial peptides (Walker and Iyengar 2015; Figueroa‐Lozano and Vos 2019). Due to the immature gastrointestinal tract of newborns, mild digestive problems, such as inefficient digestion and impaired absorption of proteins, lipids, and lactose, as well as gut dysbiosis, are often seen in infancy. The differences in composition between infant formula and human milk make mild digestive problems more likely to occur in formula‐fed infants (Jiang et al. 2022). A healthy gut barrier is essential for preventing the development of gastrointestinal disorders, autoimmune diseases, and systemic inflammation, highlighting the importance of breastfeeding in promoting optimal gut health (Vieira Borba et al. 2018).

The beneficial effects of breastfeeding on gut microbiota development and gut health extend beyond infancy and have long‐term implications for overall health and disease risk later in life. Several studies have shown that breastfeeding during infancy is associated with a reduced risk of developing gastrointestinal disorders, such as irritable bowel syndrome, celiac disease, and inflammatory bowel disease, in childhood and adulthood (Kamphorst et al. 2021; Torun et al. 2021; Bertin et al. 2023). Furthermore, emerging evidence suggests that early‐life gut microbiota composition may influence metabolic health, immune function, and neurological development, potentially impacting the risk of chronic diseases, such as obesity, diabetes, and neurodevelopmental disorders in later life (Sarkar et al. 2021; Daliry and Pereira 2021).

### 6.5 The Role of Maternal Milk in Infant Cognitive Development

The nutritional composition of breast milk is uniquely tailored to meet the evolving needs of the growing infant, supporting not only physical growth but also cognitive and neurological development (Sánchez et al. 2021; De‐Weerth et al. 2023). Research has shown that breast milk contains high levels of long‐chain polyunsaturated fatty acids, particularly docosahexaenoic acid and arachidonic acid, which are critical for brain development and function (Carlson and Colombo 2016; Basak et al. 2021). These long‐chain polyunsaturated fatty acids play a vital role in neuronal membrane structure, synaptogenesis, and neurotransmission, thereby influencing cognitive function, learning, and memory (Basak et al. 2021; Khor 2022). Research has shown that infants fed with breast milk, which naturally contains milk fat globule membrane, experience numerous health benefits compared with those fed with formula. These benefits include reduced risk of infections, improved cognitive development, and better overall growth and development (Hernell et al. 2016). In recent years, there has been growing interest in incorporating milk fat globule membrane into infant formulas to mimic the composition of breast milk more closely. Formula‐fed infants supplemented with milk fat globule membrane have been shown to exhibit developmental outcomes similar to those of breastfed infants, indicating the potential of milk fat globule membrane supplementation in promoting infant health and development (Timby et al. 2015).

Breastfeeding has been associated with improved neurodevelopmental outcomes in infants, including better cognitive performance, language development, and academic achievement later in childhood and adolescence (Anderson, Johnstone, and Remley 2016; Mohammed et al. 2022). A prospective birth cohort study found that breastfeeding was positively associated with intelligence quotient scores, language development, and academic performance in children and adolescents (Kim and Choi 2020). The bioactive compounds present in breast milk, such as growth factors, hormones, and cytokines, play a crucial role in supporting neurodevelopment by promoting neuronal growth, myelination, and synaptic plasticity (Carlson and Colombo 2016; Sambra et al. 2021). These bioactive components also contribute to neuroprotection, reducing the risk of neurodevelopmental disorders and cognitive impairments in later life (Binns and Lee 2019; Anderson, Vaillancourt, et al. 2016; Guzzardi et al. 2020). For instance, multiple sclerosis is a dynamic neuro‐inflammatory disease characterized by the accrual of multifocal areas of demyelination within the central nervous system. Infant breastfeeding appears protective against some childhood multiple sclerosis (Brenton et al. 2017).

The cognitive benefits of breastfeeding extend into childhood and adolescence, with long‐term implications for academic achievement, cognitive abilities, and socioeconomic success in adulthood (Purkiewicz et al. 2025). Several epidemiological studies have demonstrated that breastfeeding duration and exclusivity during infancy are associated with higher intelligence quotient scores, better academic performance, and increased educational attainment later in life (Victora et al. 2015; Sajjad et al. 2015). The enhanced cognitive stimulation, socioemotional support, and neurodevelopmental advantages provided by breastfeeding contribute to the development of cognitive skills, problem‐solving abilities, and academic readiness, which are essential for success in school and future endeavors (Mohammed et al. 2022; Muktamath et al. 2023).

### 6.6 Reducing the Risk of Neurodevelopmental Disorder

Attention deficit/hyperactivity disorder and autism spectrum disorder are both complex neurodevelopmental conditions characterized by specific behavioral patterns. Attention deficit/hyperactivity disorder is characterized by symptoms of inattention, impulsivity, and hyperactivity (Gleason and Humphreys 2016). Multiple studies have investigated the potential protective effects of breastfeeding against the development of attention deficit/hyperactivity disorder. A systematic review and meta‐analysis examined two cohort studies, seven case–control studies, and three cross‐sectional studies involving 3686 cases and 106,907 participants and found that breastfeeding was associated with a reduced risk of attention deficit/hyperactivity disorder. Another study revealed that children with attention deficit/hyperactivity disorder had significantly less breastfeeding duration (Tseng et al. 2019). The protective effects of breastfeeding against attention deficit/hyperactivity disorder may be attributed to various factors. Breast milk contains essential nutrients and bioactive compounds that support optimal brain development, including long‐chain polyunsaturated fatty acids, which are crucial for cognitive and behavioral functions (Gleason and Humphreys 2016; Innis 2014). Additionally, the bonding and interaction between the mother and infant during breastfeeding may contribute to the child's social and emotional development, potentially reducing the risk of attention deficit/hyperactivity disorder (Victora et al. 2015; Sajjad et al. 2015; Zeng et al. 2020).

Autism spectrum disorder is defined by difficulties in social communication and repetitive behaviors (Tseng et al. 2019). Emerging evidence suggests that breastfeeding may play a role in reducing the risk of autism spectrum disorder. A systematic review, dose–response analysis, and meta‐analysis discovered that breastfeeding exhibits a protective influence against autism spectrum disorder risk, with exclusive breastfeeding showing the lowest risk, indicating a dose‐dependent effect (Ghozy et al. 2020). The potential protective effects of breastfeeding against autism spectrum disorder are thought to be linked to the immunological and neuroprotective properties of breast milk. Breast milk contains antibodies, hormones, and other bioactive components that support the infant's immune system and protect against infections and inflammation, which have been implicated in the pathogenesis of autism spectrum disorder (Husk and Keim 2015; Soke et al. 2019). Furthermore, breast milk provides essential nutrients and growth factors that are essential for brain development and function, potentially reducing the risk of neurodevelopmental disorders like autism spectrum disorder (Martins et al. 2020; Martinat et al. 2021). However, it is important to note that although breastfeeding may reduce the risk of attention deficit/hyperactivity disorder and autism spectrum disorder, it is not a guarantee against these conditions. Genetic and environmental factors also play significant roles in the development of attention deficit/hyperactivity disorder and autism spectrum disorder. Therefore, further research is needed to better understand the mechanisms underlying the relationship between breastfeeding and neurodevelopmental outcomes and to explore potential strategies for optimizing breastfeeding practices to maximize its benefits (Yenkoyan et al. 2024).

### 6.7 Reducing the Risk of Progressive Neurodegenerative Disorders

Alzheimer's disease is a progressive neurodegenerative disorder characterized by cognitive decline, memory loss, and impaired daily functioning (Breijyeh and Karaman 2020). Breast milk is a complex fluid containing a myriad of bioactive components, including essential fatty acids, antioxidants, growth factors, and immune modulators, which are crucial for optimal brain development and function in infants (Andreas et al. 2015; Yadav et al. 2022). It is rich in omega‐3 and omega‐6 fatty acids, particularly docosahexaenoic acid and arachidonic acid, which play a pivotal role in neuronal growth, synaptogenesis, and cognitive function (Carlson and Colombo 2016; Sambra et al. 2021). It also contains antioxidants and growth factors, such as brain‐derived neurotrophic factor and IGF, which exert neuroprotective effects and promote neuronal survival and plasticity (Chen et al. 2020; Atkinson and Dickman 2023). Breastfeeding has been shown to enhance synaptic connectivity and neural plasticity, which are fundamental mechanisms underlying learning, memory, and adaptive cognitive function throughout the lifespan (Kim et al. 2016; Polverino et al. 2021). Consequently, a protective effect of breastfeeding is associated with the reduced risk of Alzheimer's disease, suggesting that longer duration of breastfeeding may confer a protective effect against Alzheimer's later in life. The neuroprotective and cognitive‐enhancing effects of breastfeeding, mediated through essential fatty acids, antioxidants, and growth factors, may contribute to reduced neuroinflammation, oxidative stress, and amyloid‐beta accumulation, which are hallmark pathological features of Alzheimer's disease (Binns and Lee 2019; Anderson, Vaillancourt, et al. 2016; Guzzardi et al. 2020).

### 6.8 Nurturing Psychosocial Well‐Being in Infancy

Breastfeeding is not only essential for an infant's physical health but also has significant implications for their psychosocial development. The act of breastfeeding fosters a unique bond between mother and baby, providing emotional nourishment alongside the nutritional benefits of breast milk (Ford et al. 2020; Lawrence 2022). Breastfeeding promotes a strong bond between mother and baby, facilitating the development of secure attachment relationships. Skin‐to‐skin contact, eye contact, and close physical proximity during breastfeeding stimulate the release of oxytocin, which plays a crucial role in maternal–infant bonding (Innis 2014; Ivanova et al. 2021). Research has shown that infants who are breastfed tend to exhibit more secure attachment behaviors, better emotional regulation, and increased maternal responsiveness compared with formula‐fed infants (Ventura 2017; Keim et al. 2021). Secure attachment lays the foundation for healthy psychosocial development, promoting resilience, self‐esteem, and positive interpersonal relationships throughout life (Modak et al. 2023). The bonding and attachment that occur during breastfeeding foster a secure and nurturing environment, which is essential for emotional regulation, social skills, and overall well‐being (Modak et al. 2023).

Breastfeeding provides infants with a comforting and soothing experience, helping them to develop effective emotional regulation skills and stress resilience. The close physical contact, rhythmic sucking, and gentle rocking associated with breastfeeding have a calming effect on infants, reducing cortisol levels and promoting relaxation (Uvnas‐Moberg et al. 2019). Studies have demonstrated that breastfeeding is associated with better emotional self‐regulation, reduced stress reactivity, and lower rates of anxiety, depression, and behavioral problems in children and adolescents (Modak et al. 2023; Turner et al. 2019; Rochat et al. 2016; Hernández‐Vásquez et al. 2022). The secure attachment and emotional support provided by breastfeeding contribute to the development of coping strategies, emotional resilience, and adaptive stress responses, which are essential for psychosocial well‐being (Vesel et al. 2015).

## 7 The Multifaceted Benefits of Breastfeeding for Mother

**FIGURE 4:**
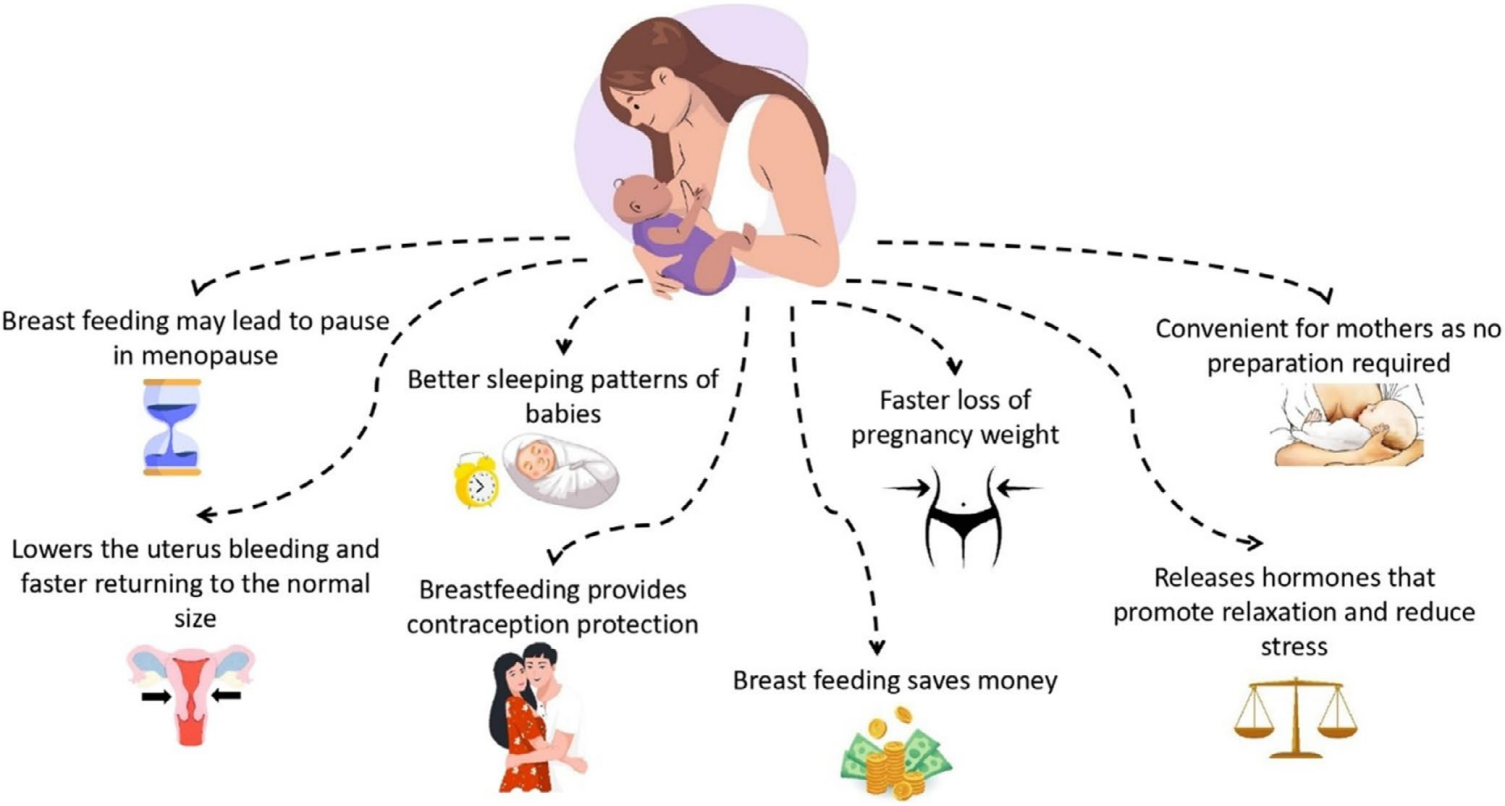
Benefits of breastfeeding for mothers.

Breastfeeding, often heralded as nature's most perfect food for infants, offers an array of benefits that extend beyond infant nutrition. As illustrated in Figure 4, the act of breastfeeding is deeply intertwined with maternal health, offering physical, psychological, and socioeconomic advantages to mothers. Breastfeeding is cost‐effective compared with formula feeding, as it eliminates the need for purchasing formula, bottles, and other feeding supplies (Breij et al. 2018). Additionally, breast milk is always available at the right temperature and does not require preparation, making breastfeeding convenient for mothers (Jersey et al. 2017). Breastfeeding promotes bonding between the mother and the baby through skin‐to‐skin contact and the release of oxytocin, a hormone associated with maternal behavior and attachment (Victora et al. 2016; Martín‐Rodríguez et al. 2022). Breastfeeding releases hormones that promote relaxation and reduce stress, contributing to improved emotional well‐being and mental health for mothers (Phillipps et al. 2020; Brahm and Valdes 2017). The psychological and cognitive impacts of breastfeeding on maternal mental health, cognitive function, and well‐being remain areas of active investigation (Purkiewicz et al. 2025). Understanding the potential cognitive benefits of breastfeeding, such as improved memory, attention, and executive function, as well as its role in promoting maternal mental health and resilience, could have profound implications for supporting maternal well‐being and optimizing parenting outcomes.

Breastfeeding accelerates postpartum weight loss by utilizing stored fat reserves accumulated during pregnancy for milk production (Breij et al. 2018). This natural weight loss mechanism aids in reducing the risk of obesity and associated metabolic disorders in mothers (Jersey et al. 2017; Ciampo and Ciampo 2018). Breastfeeding may reduce the risk of certain health conditions in mothers, such as type 2 diabetes, rheumatoid arthritis, and cardiovascular disease (Chowdhury et al. 2015; Herskin et al. 2016; Shub et al. 2019). The metabolic benefits of breastfeeding, such as improved glucose metabolism, insulin sensitivity, and lipid profile, contribute to a lower incidence of type 2 diabetes and cardiovascular risk factors in breastfeeding mothers (Pathirana et al. 2022). Lactation may play a protective role against the progression to type 2 diabetes mellitus in mothers with a history of gestational diabetes mellitus (Ma et al. 2020). Moreover, prolonged breastfeeding has been associated with improved glucose metabolism, increased insulin sensitivity, and reduced visceral adiposity, which are beneficial in mitigating the risk of type 2 diabetes (Pathirana et al. 2022).

Breastfeeding has emerged as a significant factor in reducing the risk of hypertension and cardiovascular disease among women, according to several notable studies. Park and Choi (2018) conducted a prospective cohort study published in the American Journal of Hypertension, revealing a notable finding: women who breastfed for a duration of 12 months or more exhibited a substantially decreased risk of hypertension compared with those who did not breastfeed at all. This insight suggests a tangible benefit of breastfeeding in compared with mitigating maternal health risks. Expanding upon this, Kirkegaard et al. (2018) contributed to the discourse by highlighting the importance of both full and partial breastfeeding. Their research indicated that additional partial breastfeeding demonstrated comparable significance to additional full breastfeeding in diminishing the risk of hypertension and cardiovascular disease. This underscores the holistic impact of breastfeeding duration on maternal health outcomes. Further underscoring the long‐term benefits, Peters et al. (2017) delved into the association between breastfeeding history and the risk of cardiovascular disease among Chinese women. Their findings revealed a noteworthy correlation: a history of breastfeeding corresponded to approximately a 10% reduction in the risk of cardiovascular disease later in life. Additionally, the study noted a stronger inverse relationship between breastfeeding duration and cardiovascular disease risk, emphasizing the cumulative advantages of sustained breastfeeding practices. These studies collectively reinforce the notion that prolonged breastfeeding is linked to a decreased likelihood of hypertension and cardiovascular disease among women, irrespective of prepregnancy factors, such as BMI and abdominal adiposity. By elucidating the enduring benefits of breastfeeding on maternal health, these findings underscore the importance of promoting and supporting breastfeeding initiatives for the holistic well‐being of mothers.

Numerous studies have demonstrated that breastfeeding reduces the risk of developing breast and ovarian cancers. The protective effect is attributed to the cumulative antiproliferative and differentiation‐inducing effects of breastfeeding on breast cells, as well as the interruption of ovulation during lactation (Brahm and Valdes 2017; Penacoba and Catala 2019). Breastfeeding stimulates the release of oxytocin, which facilitates uterine contractions and accelerates postpartum uterine involution, reducing the risk of postpartum hemorrhage and promoting quicker recovery (Ciampo and Ciampo 2018; Phillipps et al. 2020). Lochia is the vaginal discharge experienced by women after childbirth, which consists of blood, mucus, and uterine tissue. Breastfeeding mothers often experience a shorter duration of lochia due to the enhanced uterine contraction and involution facilitated by breastfeeding (Shao et al. 2021). Exclusive breastfeeding can act as a natural form of contraception, known as the lactational amenorrhea method, by suppressing ovulation (Tiwari et al. 2018). Furthermore, breastfeeding delays the return of menstruation, which can help protect against postpartum anemia by conserving iron stores in the mother's body (Prior 2020). Emerging research suggests that breastfeeding may have epigenetic effects, influencing gene expression patterns in mothers and potentially impacting long‐term health outcomes (Ozkan et al. 2020). Investigating the epigenetic changes associated with breastfeeding could provide valuable insights into the molecular mechanisms underlying the observed health benefits and may uncover novel pathways through which breastfeeding influences maternal health.

Breastfeeding is not only beneficial for the infant's brain health but also for the long‐term well‐being of the mother. Research by Shukri et al. (2019) suggests that breastfeeding can have lasting effects on various biopsychosocial systems related to brain health. Molly et al. (2021) further explored this by assessing how a woman's breastfeeding history correlates with her cognitive performance in postmenopausal years. Surprisingly, their findings indicate that women who breastfed perform better on cognitive tests later in life than those who did not breastfeed. However, it is important to note potential risks associated with extended breastfeeding. Studies like those conducted by Park (2017) and Ham and Bae (2023) suggest a higher risk of osteoarthritis in women who breastfeed for longer durations. Interestingly, breastfeeding's impact on postmenopausal osteoporosis appears to be nuanced. Kovacs and Ralston (2015) and Yun et al. (2016) propose that although breastfeeding may increase the risk of osteoporosis in postmenopausal women, this risk may not be definitive in individuals with sufficient vitamin D levels and calcium intakes. Therefore, maintaining adequate calcium intake and ensuring optimal vitamin D levels may play a crucial role in preventing postmenopausal osteoporosis linked to breastfeeding. Thus, to mitigate osteoarthritis in women who breastfeed, it is crucial for breastfeeding mothers to ensure they consume a diet rich in highly bioavailable calcium and other essential nutrients.

## 8 Sociocultural, Workplace, and Health Factors That Act as Barriers to Breastfeeding

Breastfeeding, being a natural and beneficial practice for both mother and child, faces numerous barriers that inhibit its prevalence and success. Breastfeeding, despite its numerous benefits, faces multifaceted barriers rooted in sociocultural, workplace, and health factors. Addressing these barriers requires a comprehensive approach involving policy changes, public education, workplace reforms, and improved healthcare support. By understanding and mitigating the following barriers, society can create an environment that promotes and supports breastfeeding as the optimal feeding method for infants, ensuring better health outcomes for both mother and child.

### 8.1 Sociocultural Factors

Sociocultural factors encompass beliefs, traditions, and societal norms that influence an individual's decision and ability to breastfeed. In many cultures, breastfeeding is not only encouraged but also deeply established as the normative feeding method for infants (Victora et al. 2016). However, certain sociocultural practices and beliefs can act as barriers. For instance, in some societies, there is a prevailing misconception that formula feeding is superior or more modern than breastfeeding (Rollins et al. 2016). Moreover, societal pressures related to modesty and the sexualization of breasts can discourage women from breastfeeding in public or even in the presence of family members (Brown et al. 2016). Such sociocultural attitudes can undermine a mother's confidence and comfort in breastfeeding, leading to premature weaning or opting for formula feeding.

### 8.2 Workplace Factors

The workplace environment plays a significant role in a mother's ability to breastfeed successfully. Many working mothers face challenges due to inadequate maternity leave policies, lack of breastfeeding breaks, and insufficient facilities to express and store breast milk (Heymann and McNeill 2016). A study by Hawkins (2023) highlighted that women who returned to work within 6 weeks postpartum were less likely to continue breastfeeding exclusively. This is primarily because the demands and constraints of the workplace make it difficult for mothers to maintain a consistent breastfeeding routine. Furthermore, the absence of supportive workplace policies and a lack of understanding from employers can create an environment where mothers feel compelled to prioritize work over breastfeeding, leading to early cessation of breastfeeding (Dagher et al. 2016).

### 8.3 Health Factors

Health factors encompass a range of maternal and infant health conditions that can present challenges to breastfeeding. Maternal health issues, such as breast surgeries, infections, and certain medications, can adversely affect milk production and quality (Wambach and Riordan 2015). Additionally, in situations where a mother dies during childbirth, breastfeeding becomes unfeasible. Furthermore, infants born prematurely or with specific health conditions may encounter difficulties in latching and effectively extracting milk (Rollins et al. 2016; Woodman et al. 2018). Insufficient milk supply is a prevalent challenge faced by some breastfeeding mothers. Despite attempts to establish lactation through appropriate nutrition and breastfeeding techniques, some mothers may struggle to produce enough milk to meet their infant's nutritional requirements (Wambach and Riordan 2015; Heymann and McNeill 2016). In these instances, supplementing with infant formula, donor human milk, or transitioning to formula feeding may become necessary to ensure the infant receives adequate nutrition. Such health‐related challenges can pose obstacles for mothers, complicating the breastfeeding process and making it less sustainable (Rollins et al. 2016).

Moreover, nipple soreness during the initial weeks of breastfeeding is a common hurdle for many mothers, often stemming from factors like improper latching, positioning, or issues with the baby's mouth (Huggins 2022). Recognizing and addressing these challenges early on is vital for fostering a positive breastfeeding journey. Engaging the expertise of a lactation consultant or healthcare provider can be invaluable in pinpointing and resolving these issues. Through appropriate guidance and techniques, they can help refine latching and positioning, easing discomfort and facilitating effective milk transfer. Self‐care is equally important during this period (Houser 2022). Utilizing soothing remedies like lanolin cream can provide relief, while allowing nipples to air dry and avoiding harsh products aids in healing (Shanazi et al. 2015; Finkelstein et al. 2018). In cases where soreness persists or concerns arise about milk supply, considering alternatives, such as feeding pumped milk or formula can offer a temporary solution (Huggins 2022; Amir and Livingstone 2016). However, seeking professional support remains paramount. Healthcare providers and lactation consultants offer personalized assistance, ensuring both mother and baby navigate breastfeeding with confidence and comfort.

Common breastfeeding insufficiencies encountered at the start of breastfeeding encompass a spectrum of challenges that can impede the successful establishment of breastfeeding (Babakazo et al. 2022). Latching difficulties pose a significant hurdle, affecting milk transfer and causing discomfort for both mother and baby. Engorgement and low milk supply are often observed, leading to inadequate milk production and potential nutritional deficits for the infant. Nipple pain and damage further hinder breastfeeding initiation and can exacerbate maternal discomfort (Noble and Rosen‐Carole 2022). Additionally, mastitis and plugged ducts present risks of breast inflammation and infection if left unaddressed. Infant‐feeding difficulties, such as weak suckling reflexes or fussiness, disrupt breastfeeding sessions and may exacerbate maternal stress (Westerfield et al. 2018). Maternal fatigue and stress can further impact milk production and the overall breastfeeding experience, highlighting the importance of addressing maternal well‐being. Insufficient milk intake in breastfed newborns is a common yet frequently overlooked issue, contributing to preventable hospitalizations for conditions like jaundice, dehydration, and hypoglycemia (Wilde 2021; Noble and Rosen‐Carole 2022). These consequences of inadequate nutrition and hydration in infancy can have long‐term neurodevelopmental implications, including conditions, such as attention deficit hyperactivity disorder, autism, and cognitive delays (Wilde 2021). Addressing these challenges requires early and adequate interventions, often involving formula supplementation or wet nursing to ensure infants receive essential nutrition and hydration (Noble and Rosen‐Carole 2022). Wet nursing, an age‐old practice, involves a lactating woman breastfeeding a child who is not her biological offspring. This practice has historical roots dating back to ancient times and has been prevalent across various cultures and societies (Vachhani et al. 2022). In contemporary contexts, wet nursing continues to offer a viable solution to breastfeeding insufficiencies, providing infants with nourishment and care when their biological mothers are unable to breastfeed due to various reasons. However, it is essential to acknowledge the cultural, societal, and logistical considerations surrounding wet nursing, as well as to ensure its ethical and practical implementation.

When breastfeeding is not recommended for certain medical problems, such as infants with classic galactosemia (Noble and Rosen‐Carole 2022; Schulpis and Iakovou 2019), alternative feeding methods must be considered. Feeding infants with metabolic illnesses (e.g., phenylketonuria) with protein‐free or modified formulae is possible but requires proper blood monitoring (Schulpis and Iakovou 2019). Mothers who test positive for human T‐cell lymphotrophic virus type I or II or have untreated brucellosis should not breastfeed or express milk to their babies (Lawrence and Robert 2021). Breastfeeding is not recommended for mothers with untreated tuberculosis or active herpes simplex lesions on their breasts (Noble and Rosen‐Carole 2022). Medical reasons often necessitate the use of infant formula when breastfeeding is contraindicated. For instance, the AAP recommends formula feeding for mothers with HIV to prevent mother‐to‐child transmission (AAP 2022). Similarly, certain medications or medical conditions may introduce risks to the infant through breast milk, making formula feeding the safer alternative (Brown et al. 2016). Women undergoing treatments such as chemotherapy or radiation therapy may face temporary or permanent challenges in breastfeeding due to potential harm posed to the infant (Mitchell and Johnson 2022). Psychological well‐being is another crucial consideration in the breastfeeding versus formula‐feeding debate. Although breastfeeding offers numerous physical and emotional benefits for both mother and baby, it can also be emotionally challenging for some mothers. Feelings of stress, anxiety, or guilt stemming from breastfeeding difficulties can negatively impact maternal mental health and overall well‐being (Shukri et al. 2019; Lau 2018). In these situations, formula feeding or donor breast milk may offer relief and alleviate some of the emotional pressure associated with breastfeeding challenges.

To effectively address the sociocultural, workplace, and health‐related barriers to breastfeeding, targeted policy interventions are essential. Hospitals should adopt evidence‐based practices, such as those outlined in WHO (2017) policy recommendations, which emphasize breastfeeding policies, ensure staff are trained, and inform expectant mothers and families. Further regulatory frameworks must also enforce stricter controls on formula marketing to prevent misleading claims and reduce early formula introduction, which has been linked to reduced breastfeeding duration. Globally focused public health organizations, including the WHO, United Nations Children's Fund (UNICEF), and the International Labour Organization (ILO) advocate comprehensive systemic reforms to support breastfeeding. These reforms encompass workplace accommodations, such as paid maternity leave, designated lactation breaks, and private pumping facilities; community‐based education initiatives to raise awareness and normalize breastfeeding; and structured training programs for healthcare providers to ensure consistent and informed breastfeeding support across diverse settings (Vilar‐Compte et al. 2021). Importantly, emerging evidence highlights the role of paternal leave in supporting breastfeeding success. Parker et al. (2025) indicate that fathers who took at least 2 weeks of leave were 31% more likely to report breastfeeding at 8 weeks postpartum compared with those who took less time. The implementation of initiatives like the Baby‐Friendly Hospital Initiative (BFHI) and Baby‐Friendly Community Initiative (BFCI) has shown success in improving breastfeeding rates when supported by strong political leadership, funding, and ongoing quality assurance (Walsh et al. 2023). These measures, when implemented collectively, can foster a supportive environment that promotes exclusive breastfeeding and improves maternal and infant health outcomes.

## 9 Advantages and Challenges of Utilizing Donor Breast Milk in Neonatal and Infant Care

Donor breast milk refers to breast milk donated by lactating mothers to milk banks or similar organizations, which is then provided to infants who are unable to receive their mother's milk (Peila et al. 2016; Senthilkumar 2018). The use of donor breast milk has several advantages, both for the infants and the healthcare system. Donor breast milk provides essential nutrients and antibodies that are crucial for the growth and development of infants (Yu et al. 2019). It contains a balanced mix of proteins, fats, carbohydrates, vitamins, and minerals, tailored to meet the nutritional needs of newborns (Yu et al. 2019; Victora et al. 2016). Donor breast milk is rich in antibodies, immune factors, and beneficial bacteria that help protect infants against infections and illnesses. This is particularly important for premature or low birth weight infants who are more susceptible to infections (Corpeleijn et al. 2016). Studies have shown that the use of donor breast milk can significantly reduce the risk of necrotizing enterocolitis, a serious gastrointestinal condition commonly seen in premature infants (Altobelli et al. 2020). The protective factors present in breast milk help promote gut health and reduce inflammation (Yu et al. 2019). Utilizing donor breast milk can be cost‐effective compared with the use of formula or specialized medical interventions required for infants with health issues. This can result in savings for families and healthcare systems in the long run (Senthilkumar 2018). For mothers who are unable to breastfeed their infants due to medical reasons or other challenges, the availability of donor breast milk can alleviate feelings of guilt or inadequacy. It provides them with an alternative option to ensure their baby receives the best possible nutrition (Senthilkumar 2018; Yu et al. 2019; Victora et al. 2016).

Although donor breast milk offers numerous benefits, its use is not without challenges. Several key issues and concerns surround the utilization of donor breast milk in neonatal care and infant feeding. The availability of donor breast milk can be limited, especially in regions where milk banks or donation programs are scarce (Orsetti et al. 2023). This can result in unequal access to donor milk for infants who could benefit from it. Ensuring the safety and quality of donor breast milk is crucial (Senthilkumar 2018). Proper screening of donors for infectious diseases and rigorous pasteurization processes are essential to minimize the risk of contamination and transmission of pathogens (Peila et al. 2016). Donor breast milk can be expensive, making it financially challenging for some families and healthcare systems. The cost of collecting, processing, and distributing donor milk can be higher than formula feeding (Senthilkumar 2018; Yu et al. 2019). In some cultures and communities, there may be stigma or misconceptions associated with the use of donor breast milk. This can lead to reluctance or resistance from families and healthcare providers to accept and promote donor milk as a viable feeding option (Peila et al. 2016; Hairston et al. 2019). Donor breast milk may have variable nutrient composition compared with mother's own milk, which can make it challenging to meet the specific nutritional needs of individual infants, particularly those with medical conditions or feeding difficulties (Sisk et al. 2017). The ethical considerations surrounding the use of donor breast milk, such as informed consent, donor anonymity, and equitable distribution, need to be carefully managed to ensure transparency and fairness in the donation and utilization process (Senthilkumar 2018).

## 10 Infant Formulas: Composition and Advancements

### 10.1 Composition of Infant Formulas Compared With Breast Milk

When breastfeeding or providing donor breast milk is not feasible, infant formulas can serve as nutritious alternatives to meet the nutritional needs of infants. Although achieving an exact replication of breast milk is challenging, significant efforts have been made to mimic its nutritional profile to meet the growth requirements of newborns (Zou et al. 2017; Ahern et al. 2019; Jiang and Guo 2021). Formulas are designed to deliver an appropriate calorie count per serving to meet the energy requirements of growing infants (Koletzko 2017). Similar to breast milk, infant formulas contain a blend of whey and casein proteins. Some formulas adjust this protein ratio to reflect the dynamic changes observed in breast milk as infants grow (Luo et al. 2023). In addition to proteins, infant formulas incorporate a blend of vegetable oils to provide essential fats necessary for growth and development. Commonly used vegetable oils include palm oil, soy oil, and coconut oil. These oils contribute to the energy density of the formula and help in the absorption of fat‐soluble vitamins (Koletzko 2017; Hageman et al. 2019). To mimic the natural presence of important nutrients in breast milk, long‐chain polyunsaturated fatty acids like docosahexaenoic acid and arachidonic acid are added to infant formulas to support brain and eye development (Luo et al. 2023; Hageman et al. 2019). Carbohydrates in infant formulas, especially in lactose‐free variants, may include ingredients like corn syrup solids or sucrose to provide energy and promote palatability (Anderson et al. 2022). Additionally, fructose must be excluded due to intolerance issues (Martin et al. 2016). Essential vitamins and minerals such as vitamins A, D, E, and K are added to infant formulas to support overall growth and development. Additionally, B‐vitamins, calcium, phosphorus, magnesium, and iron fortification address various metabolic and developmental needs of infants (Ahern et al. 2019).

Infant formulas can be broadly categorized into regular bovine milk‐based and specialized formulations tailored to meet specific dietary needs (Jiang and Guo 2021). Specialized formulas are formulated to address the unique nutritional requirements and health conditions of infants. For preterm infants, formulas may vary in their carbohydrate, protein, and fat content to support optimal growth and development (Ahern et al. 2019; Jiang and Guo 2021). Moreover, specialized infant formulas designed to mimic colostrum often contain added bioactive components, such as immunoglobulins, growth factors, and other immune‐boosting compounds, to provide enhanced immune support for newborns (Ahern et al. 2019). These formulas aim to offer some of the protective benefits of colostrum, such as improved immune system development, reduced risk of infections, and enhanced gastrointestinal health (Jiang and Guo 2021; Martin et al. 2016).

Several types of specialized formulas are available to cater to infants with specific dietary sensitivities or medical conditions. Lactose‐free formulas are designed for infants with lactose intolerance, ensuring they receive essential nutrients without experiencing digestive discomfort (Anderson et al. 2022). Hydrolyzed protein formulas break down the protein molecules into smaller fragments, reducing the risk of allergic reactions and making them suitable for infants with cow's milk protein allergy (Koletzko 2017). Soy‐based formulas provide an alternative protein source for infants with cow's milk protein allergy or those on a vegetarian diet, although they are not recommended for premature infants due to their elevated levels of phytoestrogens (Lasekan et al. 2015). Amino acid‐based formulas contain free amino acids, making them hypoallergenic and suitable for infants with severe food allergies or malabsorption issues (Borschel et al. 2018). Only L‐form amino acids are permitted, as D‐forms may lead to D‐lactic acidosis (Martin et al. 2016). Amino acid‐based formulas are particularly beneficial for treating low birth weight, premature, and critically ill infants (Jiang and Guo 2021). Moreover, for infants with allergies or sensitivities, formulas with hydrolyzed proteins or elemental formulas containing individual amino acids may be recommended (Borschel et al. 2018). In addition to these specialized formulas, some infant formulas are fortified with structured lipids designed to enhance stomach emptiness and nutrient absorption, thereby promoting better digestion and overall growth in infants (Hageman et al. 2019).

The development of the neonatal and infant intestinal microbiome is significantly influenced by factors, such as gestational age, mode of delivery, and feeding method, whether breast milk or formula. Postnatal immune maturation and the establishment of a diverse and functional intestinal microbiome are closely linked to the type of feeding, highlighting the critical role of nutrition in gut development and function (Ahern et al. 2019; Martin et al. 2016). Exclusive breastfeeding for a minimum of 6 months is well recognized for its numerous benefits compared with formula feeding (Luo et al. 2023; Bakshi et al. 2023). Breast milk not only provides essential nutrients but also plays a fundamental role in shaping the infant's intestinal microbiome and gut‐associated immune system. To replicate these beneficial effects, the composition of the latest generation of infant formulas is being optimized to mimic human milk. Innovative approaches are being employed to design new infant formulas that emulate the composition and effects of breast milk on the intestinal microbiome and gut‐associated immune system. One strategy involves the incorporation of bioactive ingredients, such as HMOs, which are complex sugars abundant in human milk that promote the growth of beneficial gut bacteria and enhance immune function (Hegar et al. 2019). Furthermore, the concept of post‐biotics, or milk fermentation products, is gaining attention. These are bioactive compounds produced during the fermentation of milk and are believed to offer similar health benefits as probiotics (Hegar et al. 2019; Fabiano et al. 2021). Additionally, to promote gut health, some infant formulas include prebiotics like fructo‐oligosaccharides (FOSs) and galacto‐oligosaccharides (GOSs), which serve as food for beneficial gut bacteria (Fabiano et al. 2021; Bakshi et al. 2023). Moreover, specialized formulas may contain probiotics and nucleotides to further support immune function, gut health, and overall growth and development in infants (Luo et al. 2023; Bakshi et al. 2023).

Despite significant advancements in the infant food industry, particularly in ensuring the adequacy of basic nutritional compounds and the incorporation of bioactive ingredients, there remains a lack of consensus regarding the functional equivalence of these novel bioactive compounds compared with those naturally present in human milk (Almeida, Mendonça Pereira, et al. 2021; Generalic Mekinic and Simat 2025). Although these innovative ingredients are intended to replicate the beneficial effects of human milk on the intestinal microbiome and gut‐associated immune system, their efficacy in infant formulas is still a subject of ongoing research and debate (Hegar et al. 2019). One of the primary challenges in formulating infant formulas with bioactive ingredients lies in preserving their bioactivity and ensuring their bioavailability post‐thermal processing and storage. Thermal treatments, such as pasteurization and sterilization, are necessary to ensure the safety and stability of infant formulas but can potentially compromise the bioactivity of sensitive bioactive compounds. These thermal processes may denature or degrade the bioactive ingredients, thereby reducing their effectiveness in providing the intended health benefits to infants (Fabiano et al. 2021; Ahern et al. 2019; Martin et al. 2016). Therefore, it is crucial to investigate the stability and retention of bioactivity of these compounds under various processing conditions to optimize their efficacy in infant formulas. Additionally, there is a pressing need for comprehensive clinical studies to evaluate the physiological efficacy of bioactive compounds already incorporated into commercially available infant formulas and those still in the experimental phase. Clinical trials are essential to assess the impact of these bioactive ingredients on infant health outcomes, including growth, immune function, and overall development. Understanding the physiological effects of these compounds in a real‐world setting is crucial to validate their safety and efficacy and to inform evidence‐based recommendations for infant‐feeding practices. Furthermore, the potential interactions between different bioactive ingredients and their synergistic or antagonistic effects on infant health should be thoroughly investigated. The complex interplay between various bioactive compounds, as well as their interactions with the infant's developing gut microbiome and immune system, necessitates a holistic approach in evaluating their physiological effects. This includes studying the optimal combinations and concentrations of bioactive ingredients to maximize their beneficial effects on infant health.

### 10.2 Advancements in Processing Technology for Infant Formula: Ensuring Safety, Quality, and Nutritional Adequacy

**FIGURE 5:**
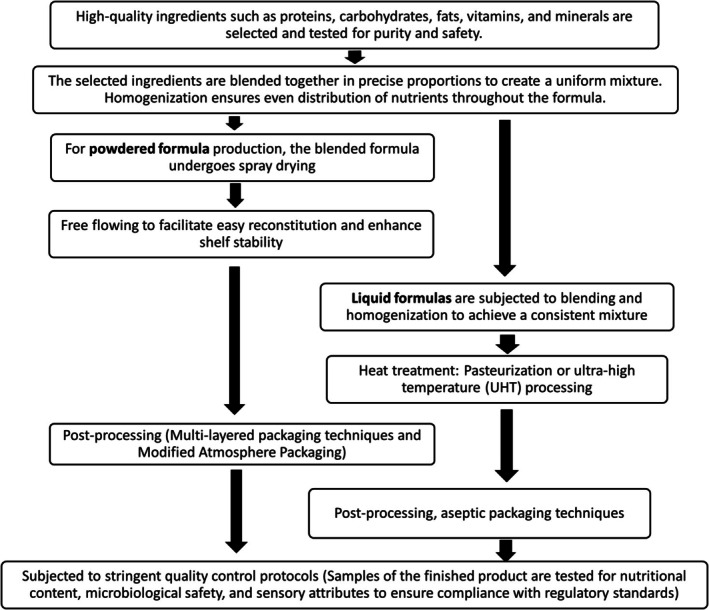
Process flow diagram of infant formula production.

Processing technology for infant formula is critical in ensuring the safety, quality, and nutritional adequacy of the final product. As illustrated in Figure 5, the manufacturing process comprises several stages, each tailored to meet specific standards and regulations to yield a product that closely replicates the composition of human breast milk. Among the various formats available, powdered and liquid infant formulas are the most commonly consumed types globally (Jiang and Guo 2021; Masum et al. 2021). It is worth noting that global rules, regulations, and quality standards governing infant formula production are crucial considerations. Regulatory bodies such as the Food and Drug Administration (FDA) in the United States and the European Food Safety Authority (EFSA) in Europe establish comprehensive guidelines and standards to safeguard the health and well‐being of infants (Hughes et al. 2017; FDA 2025). These regulations encompass various facets of production, including ingredient sourcing, manufacturing practices, labeling requirements, and quality control measures.

The initial stage in the manufacturing process involves the selection and preparation of ingredients. High‐quality raw materials, encompassing proteins, carbohydrates, fats, vitamins, and minerals, are procured from reputable suppliers to ensure both safety and nutritional quality (Hughes et al. 2017; Grote et al. 2016). Before being utilized in infant formula production, these ingredients undergo rigorous testing and screening for contaminants, allergens, and microbiological safety (Parra‐Flores et al. 2020). Once the ingredients are meticulously selected and prepared, they are blended and mixed in precise proportions to attain the desired nutritional profile and consistency (Jiang and Guo 2021). Advanced mixing technologies, such as high‐shear mixers and homogenizers, are employed to guarantee uniform distribution of ingredients, thereby eliminating any lumps or inconsistencies (Masum et al. 2021). For powdered formula production, the blended formula undergoes spray drying, a crucial step. This entails atomizing the liquid formula into fine droplets, followed by rapid drying using hot air (Jiang and Guo 2021). The resulting powder is free‐flowing, facilitating easy reconstitution and enhancing shelf stability without compromising its nutritional integrity (Jiang and Guo 2021; Masum et al. 2021). Infant milk powder formula packaging emphasizes safety, freshness, and nutritional integrity. Commonly used packaging methods include vacuum sealing, modified atmosphere packaging, tin cans, stand‐up pouches, and multilayered packaging. These techniques protect against moisture, oxygen, and contaminants, ensuring product quality (An et al. 2018).

Conversely, the production of liquid formula starts similarly with ingredient selection and preparation. Nonetheless, liquid formulas may incorporate specific ingredients to enhance solubility and stability due to their non‐powdered nature (Masum et al. 2021; Grote et al. 2016). Following this, liquid formulas are subjected to blending and homogenization to achieve a consistent mixture. High‐speed mixers and emulsifiers are often employed in this process to ensure uniform distribution of ingredients, prevent separation, and maintain a consistent texture (Jiang and Guo 2021). Heat treatment, encompassing processes like pasteurization or ultra‐high temperature processing, is imperative for liquid formula. These treatments serve to eliminate harmful pathogens while retaining the formula's nutritional content and sensory attributes (An et al. 2018). Post‐processing, aseptic packaging techniques are employed to preserve the formula's freshness and prolong its shelf life (Parra‐Flores et al. 2020). Both liquid and powdered formulas are subjected to stringent quality control protocols. Advanced testing methodologies are adopted to ensure the safety, consistency, and nutritional adequacy of the formulas (Masum et al. 2021). Moreover, optimal storage conditions, characterized by controlled temperature and humidity, are maintained to preserve the formula's quality until consumption (Hughes et al. 2017).

Whether in liquid or powdered form, infant formulas are tailored to meet the specific nutritional needs of infants at different stages of development. Stage 1 infant formula is formulated for newborns up to 6 months of age, providing higher concentrations of essential nutrients to support rapid growth and development (Grote et al. 2016; Masum et al. 2021). These Stage 1 formulas typically feature a higher whey‐to‐casein ratio, mimicking the composition of breast milk for easier digestibility and promoting softer stools. As infants grow and their nutritional requirements evolve, Stage 2 formulas are designed to accommodate these changes. These formulas adjust the whey‐to‐casein ratio to include more casein, which supports sustained energy release and growth (Baker et al. 2016). Additionally, Stage 2 formulas are fortified with increased levels of specific nutrients, such as iron, calcium, vitamin D, and vitamin C. These additions are crucial to meet the growing nutritional demands of older infants who are transitioning to solid foods and require a more diverse range of nutrients (Grote et al. 2016).

Consumer preferences for infant formula, whether liquid or powdered, are influenced by convenience, storage, preparation, and nutritional considerations. Ready‐to‐feed liquid formulas offer convenience but may have a shorter shelf life and require refrigeration (Hughes et al. 2017). In contrast, powdered formulas offer a longer shelf life and easier bulk storage but require accurate rehydration for correct nutrient concentration (Masum et al. 2021; Grote et al. 2016). Parents' choice between liquid and powdered formulas also considers infant nutritional needs and guidance from healthcare providers, with safe handling, storage, and preparation practices essential to prevent contamination and ensure safety (FDA 2025).

## 11 Nutritional and Functional Differences Between Infant Formula and Breast Milk

Infant formula may lack certain nutrients and bioactive components present in breast milk, which could affect infant development and immunity. Despite attempts to replicate human milk's composition, commercial infant formulas differ in key components, such as β‐lactoglobulin, α‐lactalbumin, lactoferrin, αs2‐casein, long‐chain polyunsaturated fatty acids, and oligosaccharides, which could impact infants' growth and development (Nguyen et al. 2015). Variations in protein composition, fat sources, and the absence of certain bioactive components may make formula milk less digestible than breast milk. Cow's milk‐based formulas often contain higher casein levels than whey, potentially leading to slower gastric emptying and digestion in some infants (Zou et al. 2017; Ahern et al. 2019; Koletzko 2017). Additionally, the fat and carbohydrate sources in formula can influence its digestibility, potentially resulting in firmer stools compared with breastfed infants (Luo et al. 2023; Hageman et al. 2019).

A study conducted in Brazil by Almeida, Baião, et al. (2021) shed light on the protein bio‐accessibility and consistency of infant formulas, revealing important insights into their nutritional quality. The study found that all tested formulas demonstrated adequate protein bio‐accessibility, indicating that the proteins were largely digested in the intestine, leading to the production of free amino acids, dipeptides, and tripeptides available for absorption. This suggests that from a digestive standpoint, infant formulas are generally effective in providing essential nutrients to infants. However, the study also uncovered concerning discrepancies regarding the total protein content among different infant formula brands. Only some brands in Brazil maintained consistent total protein content as indicated on their product labels. Moreover, variability in protein content was observed between batches from the same manufacturer. This inconsistency raises potential concerns, particularly for infants consuming the same brand regularly for extended periods, as it could impact their nutritional intake and growth. Moreover, the study by Maathuis et al. (2017) provides valuable insights into the nutritional properties and digestion kinetics of different types of infant formula. Although no significant differences were found in protein quality among the formulas tested, the similarities in digestion kinetics between goat milk‐based formula and human milk are noteworthy and warrant further investigation. These findings contribute to the understanding of infant nutrition and may have implications for formula development and optimization in the future.

The exploration of bioactive compounds in human milk, including α‐lactalbumin, lactoferrin, taurine, and others, has become a focal point for researchers in infant nutrition and food science. These compounds offer numerous health benefits, particularly their antioxidant properties, which are crucial for mitigating oxidative stress in infants, as highlighted by Almeida, Mendonça Pereira, et al. (2021). Research has revealed significant differences in the antioxidant profiles between human milk and infant formulas (Pozzo et al. 2019; Sánchez‐Hernández et al. 2021). Human milk from mothers adhering to a Mediterranean diet was found to contain a richer variety of phenolic compounds than infant formulas. Specifically, the milk from these mothers contained a higher proportion of hydroxybenzoic acids, whereas infant formulas exhibited a greater abundance of hydroxycinnamic acids. This suggests that maternal diet plays a pivotal role in determining the antioxidant composition of breast milk (Sánchez‐Hernández et al. 2021). In a study by Codini et al. (2020), the relationship between maternal diet and the fatty acid composition of breast milk was investigated. Mothers following a vegetable and fruit‐rich diet demonstrated elevated levels of total fatty acids, particularly saturated and monounsaturated fatty acids, compared with those on a Mediterranean diet. However, this increase in fatty acid content was accompanied by a decrease in antioxidant potential. Interestingly, although some infant formulas mirrored the fatty acid composition of breast milk, others displayed higher or lower antioxidant capacities. Pozzo et al. (2019) conducted a comparative analysis of the antioxidant activity and oxidative compound content of various preterm infant foods and fortifiers. Their findings indicated that human milk, whether raw or pasteurized, offered a lower intake of dietary oxidative compounds than infant formulas, especially concerning malondialdehyde content. Furthermore, the addition of fortifiers to human milk enhanced its antioxidant capacity, and the choice of protein source (hydrolyzed vs. whole proteins) influenced the overall antioxidant capacity of the diet. In recent years, there has been a concerted effort to optimize the composition of infant formulas to resemble the nutritional and functional attributes of human milk more closely. This includes the incorporation of novel bioactive ingredients with potential health benefits for infants. However, there is still a lack of consensus regarding the equivalence of these added bioactive compounds to those naturally occurring in human milk. As a result, further research is imperative to elucidate the impact of bioactive compounds on infant nutrition and to ensure the optimal health and development of infants (Almeida, Mendonça Pereira, et al. 2021; Generalic Mekinic and Simat 2025).

Human milk is a complex fluid containing not only antioxidant compounds but also a diverse array of hormones and growth factors crucial for infant development and metabolism. Research by Mazzocchi et al. (2019) and Suwaydi et al. (2021) has highlighted the presence of hormones, such as leptin, ghrelin, IGF‐1, adiponectin, and insulin in breast milk, which play key roles in hunger regulation, fat deposition, and adipose tissue metabolism throughout life. Recent efforts in infant nutrition research have focused on incorporating growth factors into infant formulas, including IGF‐1, and evaluating the safety and efficacy of adding insulin. Falcao and Zamberlan (2021) discuss ongoing investigations into the supplementation of insulin in preterm infant formulas, aiming to enhance growth and intestinal maturation in this vulnerable population. Looking ahead, future studies may explore the supplementation of insulin in term infants to further promote gut maturation and potentially prevent noncommunicable diseases, such as allergies, autoimmune disorders, and obesity. By leveraging the bioactive components found in human milk, researchers aim to develop infant formulas that more closely mimic the nutritional and developmental benefits provided by breastfeeding, thereby supporting optimal infant health and well‐being.

## 12 Health Consequences of Infant Formula Feeding

Infant formula feeding has long been a subject of debate and scrutiny within the scope of pediatric health and nutrition (Rollins et al. 2023). This controversy stems from a plethora of research findings that provide a complex picture of its potential health consequences compared with breastfeeding. Although certain studies hint at adverse outcomes associated with formula feeding when contrasted with breastfeeding, others assert the absence of significant disparities (Kazmi et al. 2023). This contradiction underscores the multifaceted nature of the issue, encapsulating various factors ranging from nutritional composition to long‐term health outcomes. Amidst these conflicting narratives, the discourse surrounding infant formula feeding remains dynamic and evolving, prompting ongoing exploration and analysis within the scientific community (Harison and Lahav 2024; Bakshi et al. 2023). Understanding the relationship between formula‐feeding practices and health outcomes is vital, as it informs healthcare providers, policymakers, and caregivers alike in making informed decisions regarding infant nutrition.

### 12.1 The Impact of Infant Formula on Gut Microbiota Composition and Infant Health

Feeding practices exert a profound influence on the composition of the gut microbiota in infants, with significant differences observed between breastfed and formula‐fed infants. Ma et al. (2020) conducted a study comparing the gut microbiota of exclusively breastfed infants with those fed different infant formulas, revealing distinct microbial profiles associated with each feeding method. Breastfed infants initially exhibited lower microbiota diversity than formula‐fed counterparts, although this increased significantly over time. Notably, breastfed infants showed higher levels of beneficial bacteria like *Bifidobacterium* and Bacteroides, whereas formula‐fed infants displayed variations depending on the formula type.

Similarly, research by Lee et al. (2015) underscored differences in gut microbiota composition between breastfed and formula‐fed infants, with breastfed infants harboring a higher proportion of probiotic species, such as *Bifidobacterium longum* , *Streptococcus salivarius* , and *Lactobacillus gasseri* . Moreover, studies by Wang et al. (2020) and Chi et al. (2021) found that *Bifidobacteria* were more abundant in the intestines of breast milk‐fed full‐term infants than in formula‐fed infants, with a decrease observed when transitioning from breast milk to formula. Conversely, potentially pathogenic genera were found to colonize more efficiently in the guts of formula‐fed full‐term infants, indicating a less favorable microbial profile compared with breastfed infants (Wang et al. 2020). Chi et al. (2021) investigated gut microbiota colonization patterns in premature infants and found that formula enriched with probiotics significantly increased beneficial bacteria like *Bifidobacterium*, whereas non‐probiotic formula led to an increase in potentially pathogenic bacteria, emphasizing that both breast milk and probiotic‐enriched formula promote the colonization of *Bifidobacteria* in preterm infants.

Furthermore, Xiao et al. (2019) demonstrated that formula‐fed infants receiving probiotics maintained higher levels of fecal secretory IgA, suggesting a positive impact on mucosal immune function. Formula‐fed infants are often more susceptible to infectious diseases due to the absence of maternal immune factors present in breast milk and the immaturity of their own immune system. Timely administration of probiotics has been proposed as a strategy to bolster immune system development in these infants. The observed disparities in gut microbiota composition between breastfed and formula‐fed infants, particularly in premature infants, have prompted interest in leveraging probiotics to modulate the infant microbiome. Studies have shown promising results, suggesting that probiotic supplementation in formula‐fed premature infants may mitigate the risk of infectious diseases and intestinal dysbiosis, such as necrotizing enterocolitis and sepsis (Nino et al. 2016; Twilhaar et al. 2018). These studies collectively reinforce the claim by showing a consistent pattern: Whereas formula feeding increases infection risk, targeted probiotic supplementation can restore immune protection and reduce disease incidence.

However, more in‐depth studies are needed to validate these findings and explore the long‐term implications of probiotic supplementation on the health and developmental outcomes of premature infants. Continued investigation in this area holds significant promise for improving the care and management of premature infants by focusing on the critical role of the gut microbiota and the potential benefits of probiotic supplementation. Future studies should aim to establish evidence‐based guidelines for probiotic supplementation in premature infants to optimize their health outcomes and reduce the risk of associated complications.

### 12.2 The Impact of Infant Formula on Childhood Allergies

Food allergies, atopic dermatitis, and asthma are prevalent childhood allergies and represent critical stages in the atopic march—a time‐based progression of allergy development affecting the skin, gastrointestinal tract, and respiratory tract (Yang et al. 2020). The development of the gut microbiota during infancy is crucial for immune system maturation. Deviations in colonization patterns have been associated with allergic manifestations, although the specific dysfunctions in the microbiome underlying allergies remain to be fully understood. The impact of infant formula feeding on the management of allergies has shown conflicting results in various studies. Kelly et al. (2019) explored the impact of formula supplementation on the likelihood of developing cow's milk protein allergy and found that breastfed babies are still at a significantly increased risk of cow's milk protein allergy by receiving supplemental formula in the first 24 h of life. This underscores the urgent need for better education of mothers and healthcare providers on the benefits of exclusive breastfeeding to minimize unnecessary formula supplementation and reduce the risk of developing cow's milk protein allergy. Contrastingly, Goldsmith et al. (2016) investigated whether infant‐feeding practices, including the duration of exclusive breastfeeding and the use of partially hydrolyzed formula, modify the risk of developing infant food allergy. The study involved 5276 infants, with 11.3% being food allergic. After adjusting for confounding variables, no association was found between the duration of exclusive breastfeeding and food allergy at 1 year of age. Additionally, the use of partially hydrolyzed formula did not reduce the risk of food allergy compared with cow's milk formula in the general population. The conflicting findings between Kelly et al. (2019) and Goldsmith et al. (2016) may be attributed to differences in study design, timing of formula introduction, and the type of formula used, particularly the inclusion of hydrolyzed proteins or other additives that can significantly influence allergic outcomes; additionally, maternal factors and feeding environments may further modulate these effects. These findings suggest that factors other than exclusive breastfeeding duration or formula type may contribute to the development of food allergies in infants.

Moreover, Wopereis et al. (2018) investigated the gut microbiota of infants at increased risk of allergy, participating in a clinical trial to assess the effectiveness of a partially hydrolyzed protein formula supplemented with nondigestible oligosaccharides in preventing eczema. The study indicated that the formula modulated the gut microbiota to more closely resemble that of breastfed infants. Additionally, a potential association between microbial activity and the onset of eczema was identified, suggesting a suboptimal implementation of gut microbiota at specific developmental stages in infants at high risk for allergy. In a similar vein, Foiles et al. (2016) demonstrated that formula enriched with long‐chain polyunsaturated fatty acids reduces the incidence of allergy in early childhood. These findings suggest that targeted nutritional interventions may beneficially modulate gut microbiota and reduce early allergic manifestations, particularly in infants at elevated risk.

Asthma is a lifelong disease with origins in early life and is the most common chronic health problem in childhood (Miliku and Azad 2018). Bigman (2020) examined the associations between breastfeeding and respiratory allergies and types of asthma in American children. This longitudinal study provided evidence that exclusive breastfeeding for the first 3 months may reduce the risk of respiratory allergies and asthma in children up to 6 years of age. Various modes of infant feeding have been associated with asthma development. Gungor et al. (2019a) suggested that moderate evidence supports the notion that feeding human milk for short durations or not at all is associated with a higher risk of childhood asthma. Conversely, Klopp et al. (2017) demonstrated that direct breastfeeding is most protective compared with formula feeding, whereas indirect breast milk confers intermediate protection. Therefore, policies that facilitate and promote direct breastfeeding could have a substantial impact on the primary prevention of asthma. Although some studies suggest potential benefits of specific formula compositions and breastfeeding practices in reducing the risk of allergies and asthma, additional investigation is needed to elucidate the underlying mechanisms and to establish evidence‐based guidelines for infant‐feeding practices to optimize immune system development and reduce the risk of associated complications.

### 12.3 The Impact of Infant Formula on Infectious Diseases

Otitis media, or ear infections, are significant causes of morbidity among infants and young children. Research indicates a strong correlation between the early introduction of formula feeding within the first 6 months of life and the escalation of acute otitis media (AL‐Nawaiseh et al. 2022). In contrast, breastfeeding has been found to reduce the risk of ear infections compared with feeding with expressed milk. Breastfed infants also exhibit better hearing outcomes compared with those who are bottle‐fed (Sequi‐Canet et al. 2020). This difference may be attributed to the mechanics of bottle feeding; when a child is bottle‐fed lying down, there is a higher likelihood of milk entering the eustachian tube, further elevating the risk of developing an ear infection (Boone et al. 2016). However, it is worth noting that specialized formulas containing bioactive compounds, such as HMOs‐supplemented formula, have shown some protective effects (Vandenplas et al. 2022). These specialized formulas not only support normal growth in infants with cow's milk protein allergy but also suggest a protective effect against respiratory and ear infections in the first year of life. Thus, although breastfeeding remains beneficial, these advanced formulas may offer an alternative for infants who cannot be breastfed, providing a potential avenue for reducing the risk of otitis media in this population.

Some research studies have highlighted that formula‐fed infants are at a higher risk of developing gastrointestinal infections, such as diarrhea and gastroenteritis, due to the lack of protective factors found in breast milk, like antibodies and probiotics (Diallo et al. 2020). Additionally, formula feeding is associated with an increased likelihood of respiratory infections, such as pneumonia and bronchiolitis, which can lead to hospitalization in severe cases (Gomez‐Acebo et al. 2021; Baker et al. 2016). However, advancements in infant formula technology have led to the development of specialized formulas that mitigate some of these risks. For instance, an infant formula supplemented with the probiotic *Bifidobacterium longum* subsp. infantis CECT7210 has been shown to reduce diarrhea episodes. This formula is safe, well‐tolerated, and associated with a lower prevalence of constipation (Escribano et al. 2018). Similarly, significant beneficial effects on infection morbidity were detected after a 3‐month intervention with bovine lactoferrin (bLF)‐fortified formula (Chen et al. 2016). These advancements indicate promising strategies for improving the safety and efficacy of infant formula, potentially reducing the risk of gastrointestinal and respiratory infections in formula‐fed infants. Nonetheless, it is crucial to continue research in this area to further refine formula compositions and ensure their safety and effectiveness in supporting infant health and development.

### 12.4 The Impact of Infant Formula on Infantile Colic

Infantile colic is a prevalent disorder, affecting between 4% and 28% of infants globally (Hjern et al. 2020). The association between feeding methods and infantile colic has been extensively studied, with some research indicating that formula‐fed infants may be at a higher risk of developing colic than of breastfed infants (Asgarshirazi et al. 2017). Infantile colic is a multifactorial condition influenced by both maternal and infant‐related factors while formula composition can also play a significant role in gastrointestinal discomfort and excessive crying in infants (Guandalini and Indrio 2024). The precise reasons for this remain unclear but may be linked to the composition of formula milk and its digestibility. Cow's milk‐based formulas differ in protein composition from breast milk, potentially contributing to digestive issues and colic in some infants (Jiang and Guo 2021; Al‐Beltagi et al. 2022). Although some studies suggest that formula‐fed infants may be more susceptible to colic than their breastfed counterparts, other research highlights that not all formulas have the same impact. Interestingly, there is emerging evidence suggesting that certain specialized infant formulas may offer benefits in managing infantile colic. Limited data indicate that using a partially hydrolyzed infant formula may be beneficial in reducing colic symptoms, particularly in formula‐fed infants without suspected cow's milk allergy (Vandenplas et al. 2016). In a study by Xinias et al. (2017), the efficacy of a lactose‐reduced synbiotic partial whey hydrolysate formula was evaluated in formula‐fed infants with infantile colic. In this study, 40 infants diagnosed with infantile colic were treated for 1 month with parental reassurance and the intervention formula (which contained partial whey hydrolysate, reduced lactose, *Bifidobacterium lactis* BB12, and GOSs). They were compared with a control group of 20 infants who received parental reassurance and a standard infant formula. Parents completed a quality‐of‐life questionnaire to assess the impact of infantile colic on their lives. The findings indicated that the intervention formula, which included partial whey hydrolysate, synbiotic components, and reduced lactose, significantly reduced the duration of crying episodes and improved the quality of life for both parents and infants. This suggests that specialized formulas with altered protein compositions and added bioactive components may play a role in managing and reducing the severity of infantile colic in formula‐fed infants. Although breastfeeding remains a primary protective factor against infantile colic, specialized infant formulas, such as partially hydrolyzed and synbiotic formulas, may offer a viable alternative for formula‐fed infants. Further research is needed to better understand the mechanisms and potential benefits of these specialized formulas in managing infantile colic effectively.

### 12.5 The Impact of Infant Formula on Risk of Development of Celiac Disease

Celiac disease is an immune‐mediated enteropathy influenced by genetic and environmental factors (Serena et al. 2019). The interaction between these factors affects disease risk. Gungor et al. (2019b) highlight limited case–control evidence indicating that feeding human milk for short durations or not at all may be associated with a higher risk of diagnosed celiac disease. The pathological process leading to gluten intolerance in celiac disease involves the intestinal microbiota, including viruses and bacteria (Ferguson et al. 2020). These components could play a role in triggering the aberrant immune response against self‐intestinal structures upon recognition of gluten peptides associated with HLA class II DQA1/DQB1 heterodimers on antigen‐presenting cells (APCs) by T cells. Both formula feeding and the HLA‐DQ2 genotype, known risk factors for celiac disease development, have been associated with an increased prevalence of pathogenic bacteria in the gut microbiota of infants at an early age (Olivares et al. 2015). This association theoretically predisposes individuals to a pro‐inflammatory gut ecosystem, potentially leading to mucosal permeability alterations and ultimately triggering celiac disease development. However, further studies are needed to establish a causal link between these risk factors and celiac disease development (Olivares et al. 2018).

### 12.6 The Impact of Infant Formula on Managing Constipation

Constipation is a prevalent issue among infants and often a source of concern for parents (Wernimont et al. 2015). A notable difference in stool patterns exists between breastfed and formula‐fed infants. Breastfed infants typically pass more frequently and have more liquid stools, whereas formula‐fed infants often have firmer stools and may experience infrequent bowel movements (Moretti et al. 2019). Human milk is associated with soft and loose stools, which is a common cause of concern for parents of formula‐fed infants, often due to its rich content of HMOs and other bioactive compounds that support gut health (Blesa‐Baviera et al. 2025; Wernimont et al. 2015). Additionally, the dynamic whey/casein ratio in breast milk, which begins with a higher whey content, enhances digestibility and contributes to softer stool consistency (Bzikowska‐Jura et al. 2018; Abdullayeva and Abdullaeva 2021). Although breastfeeding is generally associated with more favorable stool patterns, emerging research suggests that certain specialized infant formulas can effectively manage constipation in formula‐fed infants. Emerging research suggests that certain infant formulas containing bioactive compounds may offer benefits in managing constipation. A study by Benninga and Vandenplas (2019) compared the effectiveness of a magnesium‐rich formula in improving stool consistency and frequency in constipated infants. The results revealed that the magnesium‐rich formula significantly improved stool consistency and frequency compared with the control formula, suggesting its potential role in managing constipation in infants. Xinias et al. (2018) reported that a synbiotic formula containing partial whey hydrolysate and high magnesium content significantly improved functional constipation in infants. This improvement in stool consistency and frequency led to a better quality of life for both parents and infants. Wernimont et al. (2015) demonstrated that an oligofructose (OF)‐supplemented infant formula promoted the growth of bifidobacteria and resulted in softer stools without adversely affecting stool frequency or hydration. Fabrizio et al. (2022) demonstrated that whey‐dominant or hydrolyzed whey‐based formulas promote softer and more frequent stools in infants, suggesting that the whey/casein ratio is an effective component in the formulation of infant formulas to support healthy stool patterns. This suggests that prebiotic supplementation in infant formula may play a role in managing constipation by promoting beneficial gut bacteria.

### 12.7 The Impact of Infant Formula on Prevention and Management of Diseases

Formula feeding has indeed been associated with various negative effects on infant health; outcomes are often influenced by a combination of factors, including feeding practices, maternal health and nutrition, and the specific composition of the infant formula. Perrone et al. (2021) highlighted a concerning link between formula feeding and a higher incidence of sudden infant death syndrome, although the exact underlying mechanism remains unclear. This contrasts with the protective influence observed with breastfeeding and pacifier use. Additionally, Sheikh et al. (2021) identified several adverse effects associated with formula milk, including iron deficiency, low weight, gastroenteritis/diarrhea, type 1 diabetes, and autoimmune diseases. The research suggests that formula feeding may contribute to an increased risk of autoimmune diseases, such as rheumatoid arthritis and inflammatory bowel disease. Alotiby et al. (2021) found that formula feeding markedly increased the incidence of rheumatoid arthritis; although not inflammatory bowel disease, in children, highlighting the potential risk reduction associated with exclusive breastfeeding. Similarly, Lund‐Blix et al. (2017) reported a twofold increased risk of type 1 diabetes in children who were never breastfed compared with those who were breastfed. These findings underscore the significant role of breastfeeding in reducing the risk of autoimmune diseases and other adverse health outcomes in infants.

Numerous studies have highlighted the profound impact of infant‐feeding practices on long‐term health outcomes. Breastfeeding has been associated with a lower risk of chronic noncommunicable diseases, such as autoimmune conditions, cardiovascular diseases, cancer, chronic respiratory diseases, and diabetes (Cheshmeh et al. 2022). Conversely, formula‐fed infants tend to have a higher risk of developing obesity and type 2 diabetes later in life due to factors, such as formula composition and feeding practices influencing metabolic programming and appetite regulation (Uwaezuoke et al. 2017). High protein content in infant formulas has been identified as a potential contributor to rapid weight gain in the first few months of life, leading to later obesity (Lifschitz 2015). Additionally, introducing complementary foods before 4 months of age in formula‐fed or shorter‐term breastfed infants may increase the risk of childhood overweight (Pluymen et al. 2018).

The study by Cheshmeh et al. (2022) delved into the impact of different feeding methods on infant health indicators. Their findings underscored the benefits of breastfeeding, revealing that breastfed infants exhibited lower anthropometric indices and a decreased expression of obesity and diabetes‐predisposing genes compared with formula‐fed and mixed‐fed infants. This suggests that breastfeeding may confer protection against obesity and subsequent noncommunicable diseases by modulating gene expression. However, the study acknowledges the need for further investigations to elucidate the epigenetic effects of breast milk. In a similar manner, Iguacel et al. (2019) shed light on the disparities in growth trajectories between formula‐fed and breastfed infants during the complementary feeding period. Their research emphasized the protective role of breastfeeding against overweight, contrasting with formula feeding's potential to induce changes in gut microbiota associated with overweight (Forbes et al. 2018). These findings underscore the importance of breastfeeding in promoting optimal growth and development while mitigating the risk of childhood obesity and related health issues.

Although some research findings suggest negative effects of infant formula on the development of obesity and related noncommunicable diseases, there are also studies that present contradictory results. Willik et al. (2015) found that being overweight in infancy increases the likelihood of childhood overweight, regardless of whether the infant was exclusively breastfed or formula‐fed. This underscores the importance of early intervention in preventing excess weight gain from the very beginning of life, irrespective of feeding method. Moreover, some research suggests that both infant formula feeding and breastfeeding might play a role in the development of obesity to some extent. Although breastfeeding is generally associated with numerous health benefits for infants, including reduced risk of obesity later in life, certain factors, such as maternal BMI and the composition of breast milk can influence this relationship. The study by Fields et al. 2017, sheds light on the intricate relationship between human breast milk hormones and adipocytokines and infant growth and body composition during the first 6 months of life. Their data reveal that maternal BMI, infant sex, and lactation stage influence the composition of insulin and leptin in breast milk. Particularly noteworthy is the significant interaction observed between maternal BMI and infant sex on insulin levels, with obese mothers nursing female infants showing substantially higher insulin levels than normal weight mothers nursing female infants, as well as compared with obese mothers nursing male infants. It is important to note that these findings do not imply that obese mothers should refrain from breastfeeding. Rather, they provide valuable insights into the impact of maternal obesity and other factors on human breast milk composition, which remains an area of significant research interest. Understanding these dynamics may help elucidate why some studies have reported limited effects of breastfeeding on BMI in later childhood, as noted by Hancox et al. (2015).

Watchmaker et al. (2020) identified a concerning trend linking newborn overnutrition, resulting from excessive infant formula feeding on the first day of life, to an increased risk of overweight and obesity in childhood. This highlights the critical importance of appropriate feeding practices from the outset of an infant's life. In response to these findings, Ferguson et al. (2020) proposed widening the range of recommended formula amounts. By doing so, caregivers gain more flexibility to meet their infant's nutritional needs without the risk of inadvertently overfeeding. This approach promotes more balanced growth trajectories and reduces the likelihood of future overweight and obesity. This recommendation emphasizes the significance of early feeding habits in shaping long‐term health outcomes. Careful attention to feeding practices, including appropriate portion sizes, and feeding frequency, is essential for promoting optimal growth and development in infants while mitigating the risk of excessive weight gain and its associated health consequences later in life.

Gungor et al. (2019b) highlighted the need for caution in drawing conclusions about the relationship between infant milk‐feeding practices and cardiovascular disease outcomes. Although some evidence suggests that feeding less or no human milk may not be directly associated with childhood hypertension, the overall evidence remains inconclusive. Jaiswal et al. (2017) conducted a study comparing the serum lipoprotein profiles of exclusively breastfed, mixed‐fed, and formula‐fed preterm infants. Their findings revealed that breastfeeding, even when combined with mixed feeding, conferred benefits in terms of lipoprotein profiles compared with formula feeding. This suggests that breastfeeding may offer protective effects against atherosclerosis in preterm infants. Similarly, El‐Asheer et al. (2022) demonstrated a link between early infancy breastfeeding and a higher lipid profile in breastfed infants. This association suggests potential long‐term cardiovascular health benefits associated with breastfeeding that warrant support. Follow‐up studies have delved into the molecular mechanisms through which breastfeeding regulates lipid metabolism, shedding light on its role in promoting cardiovascular health. Behairy et al. (2017) observed significantly higher levels of highly sensitive C‐reactive protein in mothers and their artificially fed children compared with breastfed counterparts. However, no statistically significant difference was found in lipid profiles between the two groups. This highlights the influence of early feeding practices on the development of cardiovascular diseases, with breastfed infants and their mothers exhibiting lower highly sensitive C‐reactive protein levels, a recognized biomarker of cardiovascular disease risk. Overall, these studies underscore the importance of breastfeeding in promoting optimal lipid profiles and reducing cardiovascular disease risk in both infants and mothers. Further research into the molecular mechanisms underlying these effects is crucial for better understanding and supporting breastfeeding as a cornerstone of cardiovascular health promotion.

Verduci, Mameli, et al. (2020) proposed a mechanism where long‐term breastfeeding and delaying the introduction of solid foods until after 4 months of age, coupled with a diet rich in micronutrients, may contribute to the development of a healthy gut microbiota. This, in turn, could enhance the maturation of the immune system and potentially reduce the risk of type 1 diabetes. This hypothesis suggests a link between infant‐feeding practices, gut microbiota composition, and immune system development in the context of type 1 diabetes risk reduction. Similarly, Niinisto et al. (2017) conducted a study examining the association between early serum fatty acid composition and the risk of type 1 diabetes‐associated autoimmunity. They found differences in serum fatty acid composition between breastfed and non‐breastfed infants, with breast milk providing a distinct fatty acid profile compared with other types of milk. These fatty acids were associated with islet autoimmunity, indicating a potential role in the development of type 1 diabetes‐associated autoimmunity. Moreover, the quantity of breast milk consumed per day was inversely associated with primary insulin autoimmunity, whereas the quantity of cow's milk consumed per day was directly associated. This suggests that fatty acid status, particularly those derived from fish and consumed during breastfeeding, may confer a protective effect against the development of type 1 diabetes‐associated autoimmunity. These findings collectively suggest that infant‐feeding practices, including breastfeeding duration and the timing of solid food introduction, as well as the composition of breast milk, may influence gut microbiota, fatty acid status, and immune system maturation, ultimately impacting the risk of developing type 1 diabetes.

Juvenile idiopathic arthritis, like many autoimmune diseases, has an unknown etiology. However, Kindgren et al. (2017) found an increased risk of juvenile idiopathic arthritis in children breastfed for less than 4 months compared with those breastfed beyond 4 months, suggesting a potential protective effect of breastfeeding against juvenile idiopathic arthritis development. Similarly, Alotiby et al. (2021) highlighted the relevance of breast milk in decreasing the risk of rheumatoid arthritis compared with formula milk consumption. This underscores the potential protective role of breastfeeding in mitigating the risk of autoimmune diseases like rheumatoid arthritis. Moreover, studies have shown significant differences in gut microbiota composition between individuals with RA and healthy controls (Alotiby 2023). Breastfeeding has been associated with a protective effect against the development of inflammatory bowel diseases, with the strongest decrease in risk observed in children breastfed for up to 12 months (Ho et al. 2018; Schreiner et al. 2020). The differing microbiota profiles between breastfed and formula‐fed infants suggest that early diet‐related perturbations in the microbiome may influence disease risk. However, the protective role of human milk in autoimmune diseases remains controversial. High levels of prolactin, a hormone involved in lactation, may have varying effects, potentially leading to the development or aggravation of autoimmune diseases in susceptible mothers (Nolan et al. 2020). This raises questions about breastfeeding recommendations for mothers with autoimmune disorders, such as systemic lupus erythematosus. Thus, infant formula feeding has been associated with a potential detrimental effect against autoimmune diseases; further research is needed to fully understand the mechanisms involved and to clarify breastfeeding recommendations for mothers with autoimmune conditions.

### 12.8 The Impact of Infant Formula on Oral Health

Understanding the impact of breastfeeding and formula‐feeding on the development of dental caries in childhood is crucial for dentists, parents, and caregivers to prevent disease and shape effective public health policies. Although the issue remains complex and not fully elucidated, evidence from a meta‐analysis of cross‐sectional studies by Avila et al. (2015) suggests that breastfed children tend to be less affected by dental caries compared with those who are bottle‐fed. Similarly, findings by Rezvi and Abilasha (2016) underscore that most studies indicate a higher susceptibility to caries among children who were bottle‐fed as opposed to those who were breastfed. However, it is important to note that some studies have found no significant correlation between the two feeding methods and dental caries (Avila et al. 2015; Rezvi and Abilasha 2016). Despite these discrepancies, the prevailing consensus in current scientific literature leans toward breastfeeding offering protective benefits against early childhood dental caries. This nuanced understanding underscores the need for ongoing research and collaborative efforts among healthcare professionals and caregivers to promote optimal oral health practices in children.

### 12.9 The Impact of Infant Formula on Brain Health

Understanding the impact of infant‐feeding methods on cognitive development is crucial for caregivers and healthcare providers. Research, such as that conducted by Hou et al. (2021), suggests that breastfeeding may offer cognitive advantages. Their meta‐analysis revealed that children breastfeeding for more than 6 months exhibited slightly higher intelligence scores than those breastfed for a shorter duration. This underscores the potential influence of breast milk nutrients on neurological development, particularly in premature and term infants, as highlighted by Pang et al. (2020). Such findings emphasize the importance of supporting breastfeeding initiatives and further exploring the mechanisms through which breast milk contributes to cognitive development. Bellando et al. (2020) investigated the effects of infant feeding on childhood cognition and language, revealing small yet statistically significant differences favoring breastfeeding in verbal intelligence, expressive communication, and auditory comprehension, particularly among children aged 3 to 5 years. However, they noted that these disparities, including potential sexual dimorphic effects, may not be clinically significant. Interestingly, no significant differences were observed between cow's milk‐based and soy protein‐based formula‐fed children. Belfort et al. (2016) explored the associations between breast milk intake and neurological outcomes in very preterm infants, finding that predominant breast milk feeding in the early postnatal period was linked to better neurodevelopmental outcomes at term equivalent and 7 years of age. These outcomes included greater deep nuclear gray matter volume and improved intelligence quotient, academic achievement, working memory, and motor function. However, Von Stumm and Plomin (2015) suggested limited benefits of breastfeeding for early‐life intelligence and cognitive growth, with some evidence of a small advantage in intelligence quotient observed in girls at age 2, but not in boys, and no significant associations in later intelligence quotient growth factors. They also noted that socioeconomic status and maternal education might confound these associations. Addressing concerns about confounding factors, Horta et al. (2015a) highlighted maternal intelligence quotient as an important confounder but found that breastfeeding remained associated with enhanced performance in intelligence quotient tests even after controlling for maternal intelligence. Although numerous clinical studies suggest cognitive advantages for breastfeeding children, ongoing research is necessary to untangle the complex interplay of factors influencing cognitive development, including socioeconomic status and maternal characteristics. Nonetheless, the accumulating evidence underscores the potential long‐term benefits of breastfeeding for neurodevelopment.

Nutritional deficiencies during early life can increase susceptibility to aging‐related disorders, including cognitive decline. Essential fatty acids like docosahexaenoic acid and arachidonic acid, found in breast milk, play crucial roles in infant development (Colombo et al. 2017). However, the endogenous synthesis of these fatty acids is insufficient to maintain tissue levels equivalent to breastfed infants. Consequently, infant formulas often incorporate docosahexaenoic acid and arachidonic acid to mimic breast milk composition, aiming to support optimal development (Koletzko 2017). Intervention studies assessing docosahexaenoic acid and arachidonic acid –supplemented formulas have demonstrated various positive developmental outcomes, aligning closer to breastfed infants. These outcomes include enhanced cognitive functions, improved visual acuity, and bolstered immune responses (Lien et al. 2018). Despite these benefits, concerns have been raised regarding the significant aluminum content in infant formulas (Bondy 2016). Meta‐analyses have indicated a potential association between aluminum exposure and Alzheimer's disease, particularly with long‐term high‐concentration exposures (Corkins et al. 2019).

The impact of infant‐feeding methods on behavioral and emotional development is a significant area of research, with various studies shedding light on different aspects of this complex relationship. Poton et al. (2018) and Keim et al. (2021) suggest that formula‐fed infants may face an increased risk of behavioral and emotional problems, such as attention deficit hyperactivity disorder and depression, compared with breastfed infants. This underscores the potential influence of early nutrition on psychosocial outcomes later in life. However, Nieto‐Ruiz et al. (2020) offer a nuanced perspective by demonstrating that bioactive compound‐enriched infant formulas may have a beneficial effect on behavioral development in early childhood compared with standard infant formulas. These findings highlight the importance of formula composition in shaping behavioral outcomes and suggest that advancements in formula development could potentially mitigate some of the disparities observed between breastfed and formula‐fed infants. Moreover, sociodemographic factors, such as maternal intelligence quotient and educational level, are shown to play a significant role in child behavioral development. This further underscores the relationship between breastfeeding and psychosocial factors contributing to better mental health and fewer behavioral problems in children. Nieto‐Ruiz et al. (2020) analyzed the effects of an infant formula supplemented with specific functional nutrients on behavioral development and found similar beneficial effects compared with breastfed infants. This suggests that advancements in formula development may provide opportunities to improve behavioral outcomes in formula‐fed infants, potentially bridging the gap between breastfed and formula‐fed infants in terms of behavioral development. As new infant formulas are developed with the addition of human milk‐like bioactive compounds, including long‐chain polyunsaturated fatty acids, milk fat globule membrane, synbiotics, HMOs, sialic acid, nucleotides, or gangliosides (Poton et al. 2018; Nieto‐Ruiz et al. 2020), future studies may further elucidate the optimal composition and functionality of infant formulas to support optimal behavioral development in children. This ongoing research is essential for informing public health initiatives and guiding caregivers in making informed decisions about infant‐feeding practices.

The association between breastfeeding and the mother–child bond has been a subject of interest, but empirical evidence supporting a direct link is lacking, as noted by Penacoba and Catala (2019). Although breastfeeding has been correlated with emotional development and attachment between children and their mothers, it is essential to consider that this association may be influenced by other parenting behaviors associated with breastfeeding, as highlighted by Gibbs et al. (2018). The quality of the parent–child relationship, including mother–infant bonding, is crucial for psychological development. However, attributing a direct link between breastfeeding and bonding may stem from cultural norms rather than empirical evidence. Studies exploring this relationship have yielded inconsistent results. For example, Harrison (2019) found no significant association between breastfeeding and the quality of mother–infant bonding, nor did it mitigate the impact of mood and sleep difficulty symptoms on bonding. Although breastfeeding may offer health benefits for both mother and infant, caution is warranted when asserting its role in promoting the maternal bond. Difficulties with bonding have been reliably associated with symptoms of depression, with disrupted maternal sleep partially explaining this association, as noted by Hairston et al. (2016). These findings underscore the importance of considering multiple factors that contribute to the mother–child bond and recognizing the complex interplay between breastfeeding, maternal well‐being, and parenting behaviors in shaping this relationship.

## 13 Challenges and Barriers Associated With Infant Formula Feeding

Infant formula feeding presents a multitude of challenges and barriers that can impact families, healthcare systems, and communities. One significant challenge is the affordability and accessibility of infant formula, particularly for families with limited financial resources. The cost of formula feeding can be prohibitive, especially when not covered by healthcare or social welfare programs, placing a financial burden on already vulnerable families (Frank 2015). Furthermore, concerns about the safety, preparation, and storage of infant formula can arise, particularly in environments where access to clean water and sanitation is limited (Kent et al. 2015). Additionally, formula feeding may expose infants to environmental contaminants present in powdered formula, such as heavy metals or pesticides (Martín‐Carrasco et al. 2023). These concerns may contribute to hesitancy or reluctance to use formula feeding, even when breastfeeding is not feasible or preferred. Cultural and societal norms also play a role in shaping formula‐feeding practices, with some communities viewing formula feeding as a modern or convenient alternative to breastfeeding (Kimani‐Murage et al. 2015). These cultural perceptions may influence parental decision‐making and contribute to disparities in infant‐feeding practices. Moreover, formula feeding can interfere with natural hormonal changes triggered by breastfeeding, potentially impacting birth spacing and increasing the risk of autism and other complications in children conceived shortly after their older siblings (Gallup et al. 2016). The normalization and promotion of formula feeding by formula companies and healthcare professionals further compound these challenges. Marketing tactics aimed at promoting formula feeding may undermine breastfeeding efforts and perpetuate disparities in infant‐feeding practices (Foss 2017). Addressing these challenges requires a multifaceted approach involving comprehensive support and education for parents, healthcare providers, and policymakers. Efforts should focus on ensuring safe and appropriate formula‐feeding practices, promoting informed decision‐making about infant‐feeding options, and addressing underlying social, economic, and cultural factors that contribute to formula‐feeding disparities.

## 14 Conclusions

Breast milk is the preferred source of nourishment for infants, offering vital nutrients and adaptive immunity crucial for growth and development. For preterm or low birth weight babies, a mother's milk is ideal, with donor milk being a valuable substitute. When breastfeeding is not possible, infant formula serves as an alternative, designed to provide essential nutrients for infant health. However, formula feeding may come with some risks, including a higher incidence of gastrointestinal and respiratory issues compared with exclusive breastfeeding. Some concerns also arise about long‐term effects on metabolic health, immune function, and cognitive development due to specific formula ingredients. It is crucial to approach infant feeding as a significant health matter, transcending lifestyle choices. A collaborative effort involving healthcare professionals, policymakers, and community leaders is essential to create supportive environments for mothers, fostering inclusivity and informed decision‐making. Future research should focus on understanding the link between maternal diet and breast milk composition, exploring how dietary interventions impact milk quality and volume. Additionally, studies should evaluate the effects of specific nutritional interventions, like omega‐3 or probiotic supplementation, on breast milk and infant health. Research into the influence of maternal lifestyle factors, such as stress and sleep quality, on breast milk production is also needed. Although infant formula is a valuable alternative when breastfeeding is not feasible, caregivers must be well‐informed about its proper preparation, handling, and selection to minimize risks and ensure optimal nutrition. Pediatricians and healthcare professionals play a fundamental role in guiding caregivers on informed feeding practices, considering individual needs and circumstances. Continued research is crucial to further explore the potential health effects of infant formula, identify areas for improvement, and develop formulations that align closely with the nutritional benefits of breast milk.

## Author Contributions

**Kalmee Pramoda Kariyawasam:** writing – original draft (equal). **Geeshani Somaratne:** writing – original draft (lead), writing – review and editing (lead). **Sumali Dilrukshi Dillimuni:** writing – original draft (supporting). **Umani Walallawita:** writing – review and editing (lead).

## Conflicts of Interest

The authors declare no conflicts of interest.

## Data Availability

The authors have nothing to report.
